# Genetic and molecular signatures highlight diverse pathways linking obesity to type 2 diabetes

**DOI:** 10.1038/s41467-026-74675-9

**Published:** 2026-07-08

**Authors:** Pascal M. Mutie, Tijana Stojanovic, Valeria Lo Faro, Torgny Karlsson, Åsa Johansson

**Affiliations:** 1 Department of Immunology, Genetics, and Pathology, SciLifeLab, Uppsala University, Uppsala, Sweden; 2 Uppsala University Future Institutes (UUniFI), Uppsala University, Uppsala, Sweden

**Keywords:** Population genetics, Obesity, Type 2 diabetes

## Abstract

Obesity is a major risk factor for type 2 diabetes, a disease affecting approximately 10% of the global population. Obesity is a heterogeneous condition, with different components exerting distinct, and sometimes opposing, effects on type 2 diabetes risk. Here, we aim to identify molecular mechanisms through which distinct components of obesity influence risk for type 2 diabetes by integrating multi-omics data. We identify SNPs associated with body mass index in males and females and cluster them based on their Mendelian randomisation estimates on type 2 diabetes risk. This analysis reveals four SNP clusters with distinct effects on type 2 diabetes ranging from strongly harmful to protective. We perform cluster-specific two-sample Mendelian randomisation analyses across over 3000 molecular traits to delineate metabolic, lipid, endocrine and glycaemic pathways mediating the harmful and protective effects of distinct obesity components on type 2 diabetes risk.

## Abstract (web summary)

Obesity is a major risk factor for type 2 diabetes. Here, the authors use genetic and multi-omics data to identify molecular mechanisms linking obesity to type 2 diabetes, some with positive effects and others with negative effects on risk.

## Introduction

Type 2 diabetes (T2D) is the most common form of diabetes, affecting more than 500 million people globally, a number projected to rise to about 1.3 billion in the next two decades^1^. The rise in T2D rates coincided with the increase in obesity, with a high body mass index (BMI) being the leading risk factor accounting for more than 50% of T2D disability adjusted life years (DALYs)^1^. Further, obesity has a substantial impact on economies and households due to lost productivity related to premature mortality and sick leave, and high costs on the healthcare system, with T2D and associated cardiovascular diseases (CVD) being the largest cost drivers^2^. T2D is a heterogenous disease for which there is incomplete understanding of the causal mechanisms involved in disease onset, progression and response to treatment^3–6^. To gain a better understanding, further investigation of the processes that link established risk factors, especially excess adiposity, to T2D is warranted.

Obesity is also a heterogenous chronic disorder exhibiting distinct metabolic and phenotypic profiles with different genetic underpinnings^7,8^. Genetic findings suggest that the mechanisms contributing to obesity are diverse, as evidenced by the range of biological processes linked to BMI in genome-wide association studies (GWAS). The most recent GWAS identified 2446 genetic variants associated with BMI, representing over 900 genes, and involving interconnected disease pathways in multiple organ systems like circulatory, endocrine and nervous systems^9^. For instance, brain-related pathways involving reward and appetite control, as well as metabolic pathways tied to energy regulation, both play significant roles^10,11^. This variation highlights that multiple systems are involved in how obesity impacts health, potentially leading to distinct obesity-related conditions based on these different pathways. It is thus plausible that different pathophysiological pathways are responsible for causing obesity-related diseases.

Obesity remains the largest risk factor for T2D regardless of one’s genetic predisposition to T2D^12^. Excess adiposity is associated with insulin resistance, fatty liver, systemic inflammation and beta-cell lipotoxicity; mechanisms implicated in the causation of T2D^13,14^. Despite the current understanding, the aetiology of obesity and its role in the pathogenesis of T2D remain a topic of ongoing debate^15–17^. Previous efforts to explore the complex and heterogenous relationship between obesity and T2D have identified sets of genetic variants of adiposity associated with decreased and increased disease risk, respectively^18^. A small subset of the variants associated with obesity demonstrated a paradoxical association with cardiometabolic outcomes, showing protective effect against cardiometabolic diseases^19,20^. Most previous studies have focused on two small sets of well-studied SNPs that are strongly associated with both BMI and T2D. However, the link between adiposity and T2D is likely to be more heterogeneous than simply consisting of two clusters of genetic variants with opposite effects. Instead, multiple distinct biological mechanisms have been proposed to connect adiposity to T2D which have been described in a recent study^21^. However, the molecules that mediate such heterogeneity and characterise the molecular pathways and mechanism are still not fully elucidated.

Multi-omics traits, encompassing the plasma proteome and metabolome, as well as clinical biomarkers including glycaemic traits, provide a unique snapshot of the biological processes occurring in different organs and tissues, thereby offering valuable opportunities to deepen understanding of the pathophysiology of T2D and to advance personalised health^22^. For instance, levels of many molecules vary in different physiological states in both health and in metabolic diseases like obesity and T2D, and can also change in response to interventions like weight loss and fasting^23–26^. Studying these variations provides valuable insights into the complex pathways involved in disease onset as mediated by the involved risk factors. Further, identification of quantitative trait loci (QTLs) has facilitated the study of causal relationships between these biomarkers and cardiometabolic traits, underscoring their importance in understanding disease causal pathways^27^. To address the knowledge gap regarding the causal molecular pathways underlying the heterogeneous link between obesity and T2D, and to further characterise the mechanisms involved, we here present an innovative, data-driven multi-omics approach.

In this work, we identify distinct causal pathways mediating the effect of BMI on T2D. First, we define clusters of genetic variants representing potentially distinct biological pathways linking BMI to T2D. Second, we identify molecular traits representing the plasma proteome, plasma metabolome, and clinical biomarkers including glycaemic traits, that characterise each cluster or pathway. Third, we assess the causal impact of these molecular traits on T2D risk, thereby highlighting pathways that represent potential therapeutic targets.

## Results

**Fig. 1:**
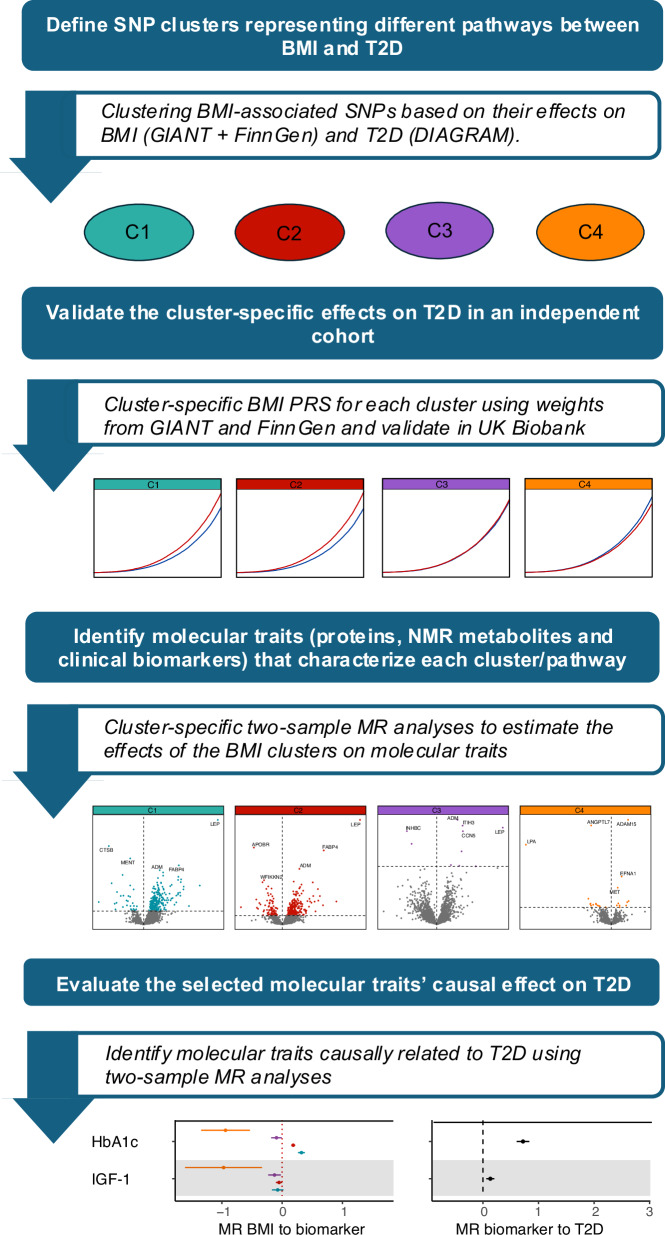
Study design overview. Abbreviations: T2D type 2 diabetes, SNP single nucleotide polymorphism, GIANT Genetic Investigation of ANthropometric Traits, DIAGRAM DIAbetes Genetics Replication And Meta-analysis, PRS polygenic risk score, MR Mendelian randomisation, BMI body mass index.

The study comprises three distinct steps making use of cutting-edge methodology and some of the largest cohorts with individual-level data and GWAS summary statistics available (Fig. 1). To identify genetic variants representing different pathways between BMI and T2D, we first clustered BMI-associated SNPs based on their effects on BMI and T2D, using a Mendelian randomisation (MR) clustering approach: MR-clust^28^. For each cluster, we validated the cluster-specific effects in an independent cohort and constructed cluster-specific BMI polygenic risk scores (BMI-PRS) to illustrate their effect on T2D and other cardiometabolic outcomes. To explore the functional relevance of these clusters, we estimated the effects of each cluster-specific BMI-SNPs on over 3000 molecular (omics) traits. Finally, we conducted MR analyses to assess the causal impact of these molecular traits on risk of T2D.

### Four clusters of SNPs potentially represent different pathways between BMI and T2D

A total of 513 independent SNPs (Supplementary Data 1) were identified as being associated with BMI in our FinnGen+GIANT meta-analysis, comprising nearly 700,000 participants. Summary statistics for these SNPs were extracted for T2D from DIAGRAM (Diabetes Genetics Replication and Meta-analysis). After MR quality control (QC) and harmonisation, 463 SNPs were retained and used to identify clusters of SNPs based on similarity in MR estimates of the effect of BMI on T2D (Supplementary Data 1).

**Fig. 2:**
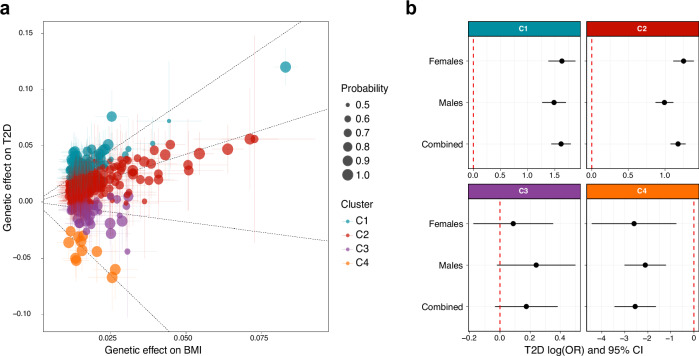
Mendelian randomisation (MR) clustering results and cluster-specific effects on type 2 diabetes (T2D). **a** Four clusters (C1, C2, C3, and C4) of BMI SNPs were identified as being differentially associated with the risk of T2D. Vertical and horizontal lines of each dot represent the 95% confidence intervals (CIs) for each SNP’s association with BMI (horizontal) and T2D (vertical). Dashed slopes represent two-sample MR estimates of the BMI-T2D causal effect, using the SNPs assigned to each cluster, separately. “Probability” refers to the likelihood of a SNP belonging to a specific cluster. **b** Validation results of the effects of the identified BMI clusters in the UK Biobank (UKB) in both sex combined (N cases = 30,737; N controls = 330,034) and sex-stratified (N cases females = 12,151; N controls females = 182,033 and N cases males = 18,586; N controls males = 148,001) analyses using two-sample MR analyses, applying the IVW method (multiplicative random-effects model). The black dots and horizontal bars represent the IVW MR point estimate and 95% confidence intervals respectively, while the dashed red vertical line represents the null effect. Colours indicate the four clusters: cyan = C1 (high-risk), red = C2 (medium-risk), lilac = C3 (null), and orange = C4 (protective). Source data are provided as a Source Data file.

We identified four clusters of BMI-SNPs that were associated with increased BMI but differentially associated with T2D (Fig. 2a), each with distinct cluster means (Table 1). Two clusters (C1-high risk and C2-medium risk) increased the risk of T2D, while one cluster (C4) had a protective effect. The remaining cluster (C3) was identified as a “null cluster”, meaning it comprised BMI-SNPs with no or very small effect on the risk of T2D. Each SNP was assigned to the cluster with the highest membership probability (Supplementary Data 1), which resulted in 85, 307, 56, and 11 SNPs being assigned to the four clusters C1-C4, respectively. Four SNPs could not be allocated to any of the other clusters and were labelled “Junk” by the algorithm and these were excluded from further analyses. This approach allowed more variants to be assigned to clusters and included in downstream analyses, compared to using a stringent probability threshold. For example, applying an 80% probability threshold resulted in very few SNPs being assigned to any cluster (Supplementary Table 1), which would have reduced our power in downstream analyses.

**Table 1:** Characteristic and MR-estimates for the four clusters identified by MR-clust

		Cluster 1 (C1)	Cluster 2 (C2)	Cluster 3 (C3)	Cluster 4 (C4)
Cluster label		High-risk	Medium-risk	Null	Protective
No. of SNPs in each cluster		85	307	56	11
Cluster means OR*	Discovery^**^	4.66	2.44	0.98	0.07
IVW MR estimates OR (95% CI)	Discovery^**^	6.17 (5.58,6.82)	2.34 (2.21,2.49)	0.70 (0.61,0.81)	0.07 (0.05,0.12)
	UKB sex-combined^***^	5.03 (4.2,6.03)	3.25 (2.93,3.60)	1.19 (0.97,1.47)	0.08 (0.03,0.19)
	UKB females^***^	5.11 (3.98,6.56)	3.50 (3.04,4.03)	1.09 (0.84,1.42)	0.07 (0.01,0.47)
	UKB males^***^	4.42 (3.55,5.50)	2.70 (2.38,3.05)	1.27 (0.98,1.65)	0.12 (0.05,0.30)

* OR corresponding to the exponentiated values of the cluster means in Fig. 2a.

** Two-sample IVW MR estimates in the discovery cohorts for respective cluster, with effect estimates for BMI from FinnGen+GIANT and effect estimates for T2D from DIAGRAM.

*** Replication cohort: IVW MR estimates for the same cluster-specific SNPs, where the SNP effects on T2D were estimated in the UK Biobank.

*IVW* Inverse variance weighted, *OR* Odds Ratio, *CI* Confidence Intervals, *UKB* UK biobank, *BMI* Body Mass Index.

Including SNPs assigned to each respective cluster, we computed two-sample MR estimates using the same cohorts. The cluster-specific inverse variance weighted (IVW) MR estimate for high-risk cluster (C1): odds ratio (OR) = 6.17 and 95% confidence interval (CI) 5.58 to 6.82 per SD increase in BMI which is slightly larger than the cluster mean (4.66) from MR-clust (Table 1). The medium-risk cluster had similar IVW MR estimate OR = 2.34 (95% CI 2.21, 2.49) as the cluster mean (2.44). In contrast, the null cluster, with a cluster mean of 0.98, had a protective causal association with T2D in the MR (IVW OR = 0.70; 0.61 to 0.81). For the protective cluster (C4), the IVW MR OR was 0.07 (95% CI: 0.05, 0.12) which was similar to the cluster mean (Table 1).

### Gene mapping and enrichment analysis of cluster-specific SNPs

To identify potential pathways represented by the different clusters, we used FUMA^29^ (functional mapping and annotation of genetic associations) to map genes to the SNPs in each cluster and perform differential gene expression analyses, as well as g:Profiler for pathway analyses^30^. The genes mapped to the high- and medium-risk clusters were enriched for genes expressed at higher levels in brain regions compared to other tissues. These regions regulate, for example, appetite (Supplementary Fig. 1a, b). For the null cluster, such enrichment was not observed to the same extent (Supplementary Fig. 1c), while the genes mapped to the protective cluster were enriched for genes expressed at lower levels in brain regions (Supplementary Fig. 1d). The risk-increasing clusters were enriched for genes annotated to, for example, biological processes including vesicle-mediated transport in synapses, neurotransmitter receptor localisation to the postsynaptic membrane, nervous system development, and differentiation of fat cells (Supplementary Data 2 and 3). In contrast, the genes mapped to the null cluster did not show strong evidence for enrichment for any pathways or GO (gene ontology) terms (Supplementary Data 4). Some of the relevant pathways for the protective cluster included response to nutrient levels, regulation of growth and muscle cell differentiation, and post-embryonic development (Supplementary Data 5).

### Validation of cluster specific estimates in UK Biobank

The effects of the four BMI-SNP clusters on T2D were validated in an independent cohort (Table 1) comprising 360,813 unrelated UK Biobank (UKB) participants of European ancestry. In this cohort, there were slightly more females (53.8%) compared to males (Supplementary Table 2). There was no statistical difference in average of age or BMI between sexes, but males had higher number (18,586 or 11.2%) of T2D diagnoses compared to females (12,151 or 6.3%).

To validate the BMI-to-T2D MR results from the previous analyses based on FinnGen+GIANT and DIAGRAM data, we estimated the effect of the 459 SNPs, representing the four clusters (excluding the 4 SNPs that were assigned to the “junk” cluster), on T2D in the UKB. We then computed two-sample IVW MR estimates for the effect of BMI on T2D for each of the four clusters, performing both a sex-combined and sex-stratified analysis. The estimates were similar to the discovery analyses; however, there was a larger effect of the medium-risk cluster in UKB (Table 1, Fig. 2b). The high- and medium-risk clusters tended to have a larger effect in females compared to males, with a significant sex-difference detected for the medium-risk cluster (P-value = 0.006).

### Disease risk associated with extremes of cluster specific BMI-PRS

**Fig. 3:**
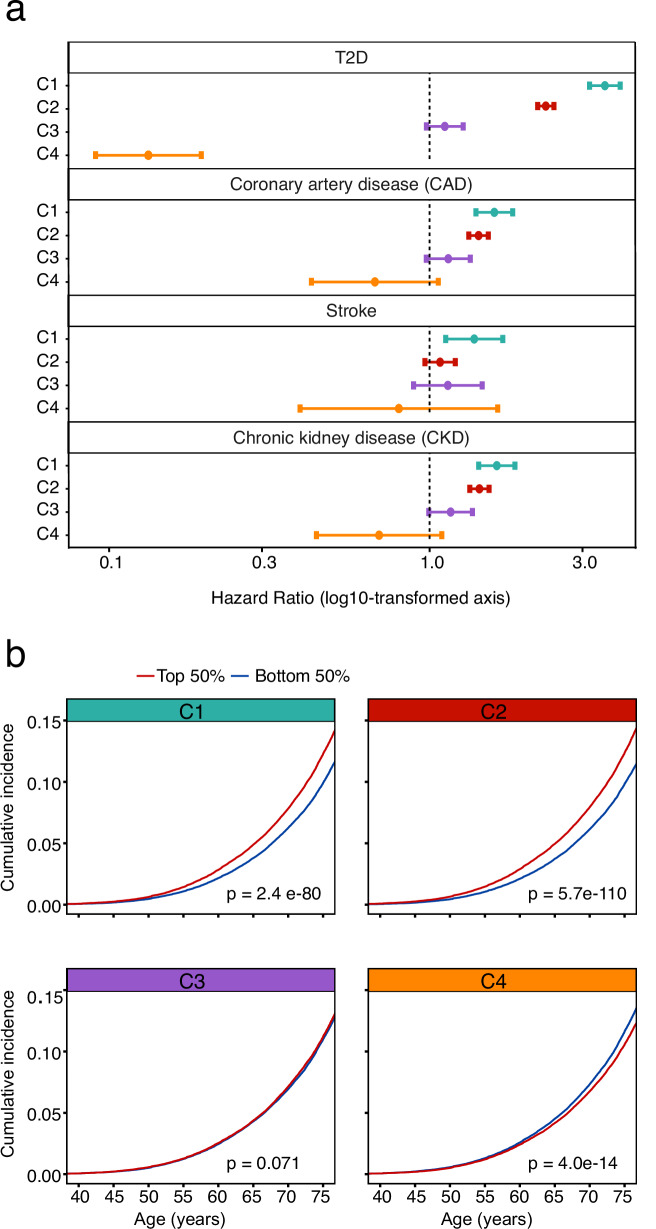
Association between PRS-BMI clusters and incidence of type 2 diabetes (T2D) in the UK Biobank (UKB). **a** Hazard ratios (HR) for four cardiometabolic outcomes associated with a 1-standard-deviation increase in BMI based on cluster-specific BMI polygenic risk scores (PRS). Dots represent HRs and horizontal bars indicate 95% confidence intervals (CIs). Estimates are derived from Cox regression models adjusted for sex and with age as the primary time scale. A total of 30,737 incident T2D cases were present among 360,771 participants, 21,945 coronary artery disease (CAD), 8,846 stroke, and 21,451 chronic kidney disease (CKD) cases. Analyses were stratified by the four clusters: cyan = C1 (high-risk), red = C2 (medium-risk), lilac = C3 (null), and orange = C4 (protective). **b** Unadjusted cumulative incidence rates of T2D among individuals in the top versus bottom 50% of each cluster-specific BMI PRS distribution. P-values are based on two-sided log-rank tests comparing the two groups; *P* < 0.05 indicates statistical significance. Colours indicate the top/bottom half of the PRS for each cluster (red = top 50% and blue bottom 50%). Source data are provided as a Source Data file.

We then extracted the effect estimates for the SNPs in the four clusters from the FinnGen+GIANT meta-analyses (Supplementary Data 1) and used these estimates to compute cluster-specific weighted BMI-PRSs in the UKB. We evaluated the effect of each cluster-specific BMI-PRS on T2D incidence in the UKB (Fig. 3a). A one standard deviation (SD) increase in BMI for the two harmful clusters (C1 and C2) was associated with a markedly increased risk of T2D (HR = 3.52, 95% CI = 3.16–3.93 and HR = 2.30, 95% CI = 2.17, 2.44, respectively). Conversely, a one SD increase in BMI due to the protective BMI-PRS was associated with a strong reduction in T2D risk (HR = 0.13, 95% CI = 0.09–0.19). Similar patterns were observed for other cardiometabolic outcomes, although differences between cluster-specific effects were less pronounced (Fig. 3a).

All clusters, in particular the protective cluster, contained a relatively small number of variants (Table 1). This means that, although the estimated effects per cluster (i.e., the change in T2D risk per 1 SD increase BMI caused by the cluster-specific BMI-PRS; Table 1 and Fig. 3a) are substantial, the limited number of SNPs does not allow to identify individuals with a full 1 SD increase in BMI attributable to the PRS of any single cluster (Supplementary Fig. 2). We therefore also examined cumulative incidence for T2D among individuals in the top 50% versus the bottom 50% of each cluster-specific BMI PRS. The difference between these groups was evident for both the harmful and the protective clusters. However, the top vs. bottom comparison for the protective cluster reflects a much smaller difference in BMI, owing to the limited number of SNPs, as compared with the two harmful clusters. This is particularly true in comparison with the medium-risk cluster, which includes more SNPs and therefore provides better discrimination between individuals (Fig. 3b).

### Cluster-specific effects on molecular traits

To elucidate potentially distinct biological pathways linking BMI to T2D across the four clusters, we aimed to identify molecular mediators that characterise these pathways. For a molecular trait to mediate the effect of a risk factor on disease, the underlying assumption is that the risk factor (high BMI) influences the mediator, which in turn affects disease risk. For each cluster, the cluster-specific BMI-associated SNPs were therefore first used as instrumental variables in MR analyses to identify molecular traits associated with each BMI cluster (Fig. 1). We further expected that difference in how the clusters influence the molecular traits would indicate that the mediator exerts distinct, cluster-dependent effects. We therefore estimated the degree of heterogeneity in the effects between the four clusters on the molecular traits using a modified chi-square test.

For these analyses, the effects of the cluster specific instruments on BMI were obtained from the FinnGen+GIANT meta-analyses described above. The corresponding effect estimates of the same SNPs on the molecular traits were mainly derived from UKB either from individual-level data or from available summary statistics datasets. We analysed three sets of molecular (omics and biomarkers) traits: 2,922 protein measures obtained from 54,219 UKB participants^31^, 249 metabolic traits quantified by nuclear magnetic resonance (NMR) spectroscopy in 619,372 individuals (UK Biobank and Estonian Biobank)^32^, and 43 biochemical traits, including 35 blood and urine biomarkers measured in the entire UKB cohort^33^, as well as 8 additional glycaemic traits obtained from the MAGIC consortium (476,326 participants with random glucose^34^; 281,416 with 2-hr, glucose, fasting glucose, HbA1c, and fasting insulin^35^; 45,861 with proinsulin^36^ and 55,000 with insulin sensitivity index and insulin fold change^37^).

Only molecular traits that were associated with at least one BMI cluster in the first MR analyses, and for which significant heterogeneity between clusters was observed (FDR < 0.05 in both steps), were considered for a second-step MR analysis to assess the potential causal effect of each molecular trait on T2D risk. For this second MR, new instruments for the respective molecular trait were identified (see Methods section) and used to evaluate their effect on T2D in data from the DIAGRAM consortium (74,124 cases, 824,006 controls). Biomarkers that remained significant in this step (IVW MR, FDR < 0.05) were subsequently evaluated in formal mediation analyses.

### Cluster-specific effects on proteins

**Fig. 4:**
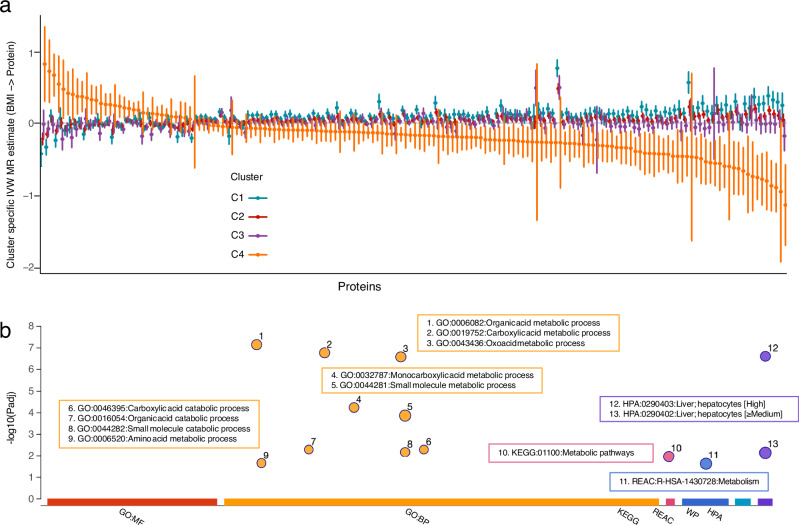
Cluster-specific effects on protein levels. **a** IVW Mendelian randomisation (MR) estimates for cluster-specific BMI to protein analyses. The centres represent the IVW MR point estimate, and the bars the 95% confidence intervals (CI). Protein measures obtained from a total of 54,219 UKB participants were included in the analysis and only proteins with significant IVW MR estimates in at least one cluster and significant heterogeneity (chi-square test) are shown. False discovery rate (FDR) adjustment was applied at 0.05 in both steps. For full information on individual proteins see Supplementary Data 7. Colours indicate the four clusters: cyan = C1 (high-risk), red = C2 (medium-risk), lilac = C3 (null), and orange = C4 (protective). **b** Enriched functional terms among proteins showing lower levels in the protective cluster and higher levels in the harmful clusters (right in panel a). Abbreviations and colour coding in enrichment analyses: red = GO:MF (Gene Ontology Molecular Function), orange = GO:BP (Biological Process), pink = KEGG (Kyoto Encyclopaedia of Genes and Genomes), blue = REAC (Reactome), cyan = WP (WikiPathways), and lilac = HPA (Human Protein Atlas). Enrichment analysis was conducted using the hypergeometric test as implemented in g:Profiler. Padj - the experiment-wide adjusted two-sided P-values which corresponds to a significance threshold of α = 0.05. Source data are provided as a Source Data file.

The cluster-specific MR analysis identified 653 proteins that were significantly (FDR < 0.05) associated with at least one cluster (Supplementary Data 6). Among these, 159 proteins showed significant heterogeneity in IVW MR estimates between clusters (FDR < 0.05), meaning that the IVW MR estimates differed significantly between clusters (Supplementary Data 7). For most of these proteins, the protective BMI cluster was associated with decreased protein levels, with a generally monotonic trend across clusters such that protein levels progressively increased from the protective to the harmful BMI clusters (Fig. 4a, Supplementary Data 7). Sex-stratified analyses did not demonstrate any differences between males and females (Supplementary Fig. 3).

Enrichment analysis identified 13 significantly enriched terms (Fig. 4b) among the proteins that were decreased in the protective and increased in the risk-increasing clusters. The results point to widespread involvement of metabolic processes, particularly those related to small molecules, organic acids, and amino acids, as well as oxidoreductase activities. A smaller number of proteins showed higher levels associated with the protective clusters (Fig. 4a), and for these, no significant terms were identified in the enrichment analyses.

Instrumental variables (IVs) were identified for 131 out of the 159 proteins with heterogeneous IVW MR estimates (see criteria in Methods). These 131 proteins were then taken forward for the second-step MR analysis (protein to T2D). However, none of the proteins reached statistical significance after multiple testing adjustment (FDR-adjusted IVW MR P-value). Among the most significant associations (unadjusted *P* = 0.00099), we identified Adiponectin (ADIPOQ), a hormone primarily secreted by adipose tissue that plays a key role in the regulation of glucose levels and fatty acid metabolism.

### Cluster-specific effects on NMR metabolites

**Fig. 5:**
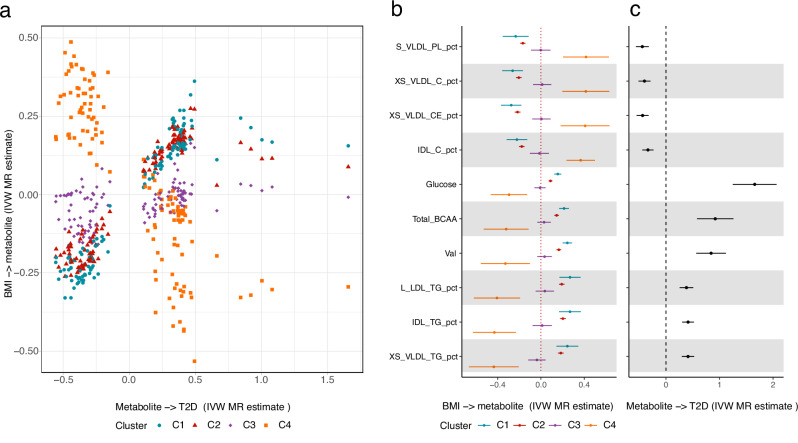
NMR Metabolites characterising potential causal links between obesity and type 2 diabetes (T2D). **a** IVW MR estimates for the effect of metabolites on T2D in relation to cluster-specific BMI IVW MR estimates on the metabolites. Only metabolites significant at FDR < 0.05 across all three steps are shown: (1) cluster-specific BMI to metabolite MR, (2) heterogeneity tests between clusters for these MR estimates, and (3) MR of metabolites to T2D. Colours indicate the four clusters: cyan = C1 (high-risk), red = C2 (medium-risk), lilac = C3 (null), and orange = C4 (protective). **b** IVW MR estimates for the effect of different BMI clusters on metabolite levels for a subset of metabolites with the most significant between-cluster heterogeneity. Dots and horizontal bars represent the IVW MR point estimates and 95% confidence intervals, respectively. These estimates were obtained from combined, meta-analysed data from the UK Biobank and Estonian Biobank (*N* = 619,372). Colours indicate the four clusters: cyan = C1 (high-risk), red = C2 (medium-risk), lilac = C3 (null), and orange = C4 (protective). **c** IVW MR estimates for the same metabolites on T2D. Dots and horizontal bars represent the IVW MR point estimates and 95% confidence intervals, respectively. T2D data were obtained from the DIAGRAM consortium (*N* = 74,124 cases and *N* = 824,006 controls). For full information and complete metabolite names, see Supplementary Fig. 4 and 5, and Supplementary Data 8 and 9. Explanations and abbreviations: Clusters: C1 = High-risk, C2 = Medium-risk, C3 = Null, C4 = Protective. Source data are provided as a Source Data file.

Out of 249 NMR metabolite traits, the vast majority (229 traits) were significantly (Supplementary Data 8) associated with at least one of the clusters in the cluster-specific MR analyses (IVW MR; FDR < 0.05). Of these, 148 also showed significant heterogeneity between clusters (chi-square test; FDR < 0.05). When assessed for their potential causal effect on T2D in a second MR step (Supplementary Data 9), as many as 133 traits showed a significant causal association (IVW MR; FDR < 0.05). Approximately half of these metabolites (*N* = 59) showed a protective effect on T2D (IVW MR estimate <0), and nearly all of these 59 metabolites were also positively associated (cluster-specific IVW MR estimate > 0) with the protective cluster (Fig. 5a, Supplementary Fig. 4, Supplementary Data 10).

This is consistent with a mechanism in which the protective BMI cluster increases the levels of these metabolites, thereby mediating a protective effect. All these metabolites were also negatively associated with the two harmful clusters (cluster-specific IVW MR estimate <0), indicating that part of the harmful effect of these clusters could operate through downregulation of metabolites that are beneficial for protecting against T2D. These metabolites define a lipid profile characterised by cholesteryl esters, cholesterol and free cholesterol fractions across HDL, LDL and multiple VLDL subclasses, together with higher phospholipid-to-lipid ratios and increased concentrations and particle diameters of large and very large HDL. The profile also includes consistently elevated ratios of omega-6 to total fatty acids, polyunsaturated to total fatty acids, and polyunsaturated to monounsaturated fatty acids, indicating a PUFA-enriched lipid milieu.

Another set of 63 metabolites showed increased risk of T2D and a consistent pattern of higher expression in the harmful and medium-risk clusters and lower expression in the protective cluster (Fig. 5). This pattern is consistent with a harmful effect by the risk-increasing clusters, mediated through increased levels of these metabolites, or a protective effect by the protective cluster mediated through their decreased levels. These metabolites (Supplementary Data 10 and Supplementary Fig. 5) define a strongly triglyceride-enriched lipoprotein profile, with marked elevations in triglyceride concentrations and triglyceride-to-total-lipid ratios across all major lipoprotein subclasses, including VLDL (very low-density lipoproteins), chylomicrons, IDL (intermediate-density lipoproteins), LDL (low-density lipoproteins), and HDL (high-density lipoproteins). The pronounced increase in total triglycerides, total lipids, and particle concentrations of small, large, and very large VLDL, as well as chylomicrons and extremely large VLDL, indicate hepatic overproduction of TG-rich lipoproteins and impaired clearance, a hallmark of insulin-resistant states. Elevated VLDL particle diameter further supports excessive hepatic secretion of large TG-rich particles. Enrichment of monounsaturated fatty acids and higher MUFA-to-total-fatty-acid ratios, combined with consistently lower phospholipid-to-lipid ratios across VLDL, HDL, and chylomicron fractions, reflect a fatty acid composition dominated by de novo lipogenesis and reduced phospholipid density.

Beyond lipid metabolism, these high-risk clusters show elevated glucose and glycoprotein acetyls, indicating impaired carbohydrate handling and heightened systemic inflammation, both tightly linked to hypertriglyceridaemia. The consistent upregulation of branched-chain amino acids (BCAA), including valine, leucine, and isoleucine as well as total BCAA concentration, and elevated alanine further support a state of insulin resistance, increased catabolic signalling, and disrupted mitochondrial substrate handling.

For the 133 NMR-derived metabolite traits identified as potential mediators in the two-step MR analyses described above, we conducted a formal mediation test. In total, 132 metabolites significantly mediated the effect of at least one BMI cluster on T2D (Supplementary Data 11). Among the top mediators was glucose, where lower levels explained 19% of the total protective effect of BMI on T2D for cluster C4, while a unit increase in glucose levels accounted for 14% and 17% of the harmful effects for the high and medium risk-increasing clusters, respectively. Similarly, mediation through total BCAA contributed between 11 and 16% to the total effects on T2D for both the protective cluster (via lower levels) and the risk-increasing clusters (via higher levels).

### Cluster-specific effects on clinical biomarkers including glycaemic traits

Out of 43 clinical biomarkers, 35 were associated with at least one BMI cluster in the IVW MR analyses (Supplementary Data 12), of which 24 showed heterogeneous effects between clusters. Seventeen of these traits (15 unique, as random glucose and HbA1c were measured in both MAGIC and UKB) were also significantly associated with T2D in the second MR analyses (Supplementary Table 3 and Supplementary Data 13). Four biomarkers, AST/ALT (Alanine to Aspartate Aminotransferase) ratio, SHBG, HDL cholesterol, and Apolipoprotein A, showed a protective effect on T2D. These biomarkers were increased by the protective BMI cluster (C4) and decreased by the risk-increasing clusters, consistent with higher levels mediating the protective effect of C4, or conversely, lower levels mediating the harmful effects of the risk clusters.

**Fig. 6:**
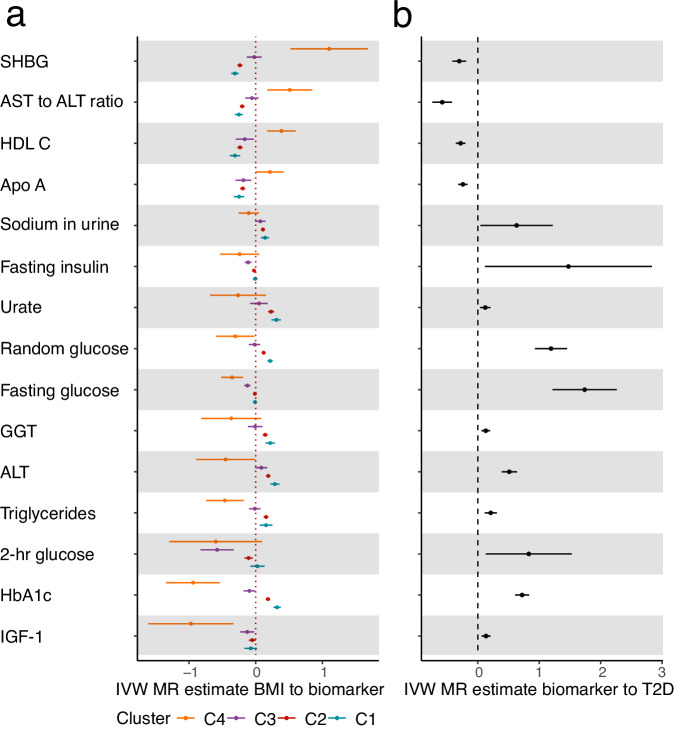
Clinical Biomarkers characterising potential causal links between obesity and type 2 diabetes (T2D). **a** Causal association between different BMI clusters and biomarker levels. Dots and horizontal bars represent the IVW MR point estimates and 95% confidence intervals, respectively. Most traits were estimated in unrelated participants self-reported as White British (*n* = 318,953), except for three traits (2-hour glucose, fasting glucose, and fasting insulin) which were measured in the MAGIC consortium (*n* = 281,416). Colours indicate the four clusters: cyan = C1 (high-risk), red = C2 (medium-risk), lilac = C3 (null), and orange = C4 (protective). **b** IVW MR causal estimates of the association between biomarkers and T2D. Dots and horizontal bars represent the IVW MR point estimates and 95% confidence intervals, respectively. T2D data were obtained from the DIAGRAM consortium (*N* = 74,124 cases and N = 824,006 controls). Explanations and abbreviations: C1 = High-risk, C2 = Medium-risk, C3 = Null, and C4 = Protective cluster. AST Aspartate Transaminase, ALT Alanine Transaminase, SHBG Sex Hormone Binding Globulin, GGT Gamma-glutamyl transferase, IGF-1 Insulin-like Growth Factor-1. For full results see Supplementary Data 13 and Supplementary Table 3. Note: In this depiction we have excluded one each of duplicated biomarkers (glucose and HbA1c) owing to availability in both UKB and MAGIC analyses. Source data are provided as a Source Data file.

The remaining 11 traits exhibited the opposite pattern; they were associated with increased T2D risk in the second-step MR analyses and negatively correlated with the protective cluster, suggesting that downregulation or lower abundance may mediate the protective effect of C4. Most of these traits were increased by the risk clusters, while a few were unaffected, indicating that upregulation of some markers may also mediate the harmful effects of the risk clusters. Among these biomarkers were well-established T2D markers such as glucose and HbA1c, as well as urate and sodium in urine, which reflect renal function, gamma-glutamyl transferase and alanine aminotransferase, markers of liver function, and IGF-1 and triglycerides, which are typical markers of metabolic and endocrine function (Fig. 6).

Out of 17 clinical biomarkers (15 unique traits), 12 were significant mediators of the effect of at least one BMI cluster on T2D in the formal mediation test. Also in these analyses, glucose mediated the largest proportion of the effect, with lower fasting glucose and random glucose accounting for 24% and 11% of the total protective effect on T2D, respectively. SHBG and the AST/ALT ratio also mediated substantial proportions of the effect (Supplementary Data 14).

### Two-sample MR to estimate the effect of BMI-clusters on imaging-derived and fat distribution traits as well as cardiometabolic diseases

In addition to molecular mediators, we investigated whether the effects of BMI on fat distribution traits and traits derived from magnetic resonance imaging (MRI) differed across clusters. The traits explored here included waist-to-hip ratio (WHR), visceral adipose tissue (VAT) mass, liver fat percentage, liver volume, pancreatic fat percentage, and pancreatic volume. Overall, we observed consistent cluster-specific effects for several traits (Supplementary Fig. 6). For fat distribution, both VAT and WHR showed trends toward more harmful patterns in the harmful clusters. However, due to the limited sample size in the imaging subset, statistical power was low and confidence intervals for the protective cluster were too wide to draw definitive conclusions. Liver and pancreatic fat percentage also tended to be higher in the harmful cluster, whereas pancreatic volume was slightly lower. These observations suggest that the most adverse metabolic clusters are associated with ectopic fat accumulation in the liver and pancreas and a non-favourable fat distribution, as well as smaller pancreatic volume.

## Discussion

We identified four clusters of BMI-associated genetic variants linked to distinct metabolites and biomarkers, representing both risk-increasing and protective pathways through which obesity influences T2D risk. Validation in an independent cohort showed that the heterogeneity of BMI effects on T2D is greater than previously recognised. While previous studies have focused on a small subset of candidate SNPs that have been assigned two groups based on their concordant and discordant BMI-T2D associations, we here present a data-driven approach where as many as four different clusters are identified. Two clusters were associated with increased T2D risk, one “null” cluster with no apparent effect, and one cluster showing a strong protective effect. However, these clusters do not represent sharply defined categories. These clusters are rather data-driven realisations of an underlying, descrete or possibly continuous, spectrum of SNPs ranging from high risk to protection. Similarly, the effects of the clusters on molecular traits followed a comparable gradual pattern: traits associated with increased T2D risk were associated with increased levels for the high-risk cluster, slightly elevated levels for the medium-risk cluster, no effect in the null cluster, and associated with decreased levels for the protective cluster. In agreement, traits protective against T2D displayed the opposite trend.

We identified 133 NMR-derived metabolites and 15 unique clinical biomarkers that were influenced by at least one BMI cluster and were, in turn, causally associated with T2D. Of these, most were also significant in the formal mediation analyses. Overall, the protective effect appears to be mediated through a more metabolically favourable profile. Central to this pattern is an HDL-associated lipid phenotype that supports efficient reverse cholesterol transport, facilitating the removal of excess cholesterol and lipids from peripheral tissues and the circulation back to the liver for excretion, thereby reducing the harmful effects of circulating lipids. These alterations indicate a shift toward cholesterol- and cholesteryl ester–enriched HDL particles with increased particle size and lipid content, alongside compositional remodelling of VLDL and LDL subclasses toward higher cholesteryl ester and cholesterol density. This lipid profile is consistent with enhanced reverse cholesterol transport, reduced triglyceride exchange, and a more anti-inflammatory and metabolically favourable lipoprotein environment. Apolipoprotein A-I, a key structural and functional component of HDL particles, further supports the presence of a cardiometabolically protective lipid milieu.

In contrast, the elevated T2D risk was mediated through a lipid and metabolic profile indicative of impaired lipid handling, reduced HDL cholesterol and insulin resistance. This adverse composition is compatible with increased cholesteryl ester transfer protein and hepatic lipase-mediated triglyceride exchange, leading to triglyceride overloading not only in VLDL, LDL, and IDL but also in HDL particles. Such remodelling promotes the formation of dysfunctional, triglyceride-rich HDL particles with reduced cardiometabolic protective capacity^38^. This pattern, characterised by extreme triglyceride enrichment, expansion of VLDL and chylomicron particles, dominance of monounsaturated fatty acids, reduced phospholipid density, elevated BCAA, systemic inflammation, and hyperglycaemia, represents a profoundly metabolically adverse lipid signature.

The increased T2D risk was also mediated by high random glucose and HbA1c. On the other hand, lower fasting glucose and fasting insulin were more strongly linked to the protective effect. This pattern suggests a dissociation between basal and postprandial glucose regulation, where preserved fasting glucose and insulin levels reflect maintained insulin sensitivity, while elevated random glucose and HbA1c indicate impaired glucose handling under metabolic stress, driving increased T2D risk^39^. This increased T2D risk was also mediated by a reduced AST-to-ALT ratio and the protective effect by increased AST-to-ALT ratio, a marker that reflects hepatic function and metabolic integrity. A similar trend was seen for SHBG, which serves as an established marker of metabolic and hormonal status^40^. Notably, high total BCAA levels, as well as the individual amino acids valine, leucine, and isoleucine, were identified as strong mediators of increased T2D risk, whereas lower levels mediated protection against T2D. This is consistent with the well-established role of BCAAs in the development of insulin resistance, potentially through enhanced catabolic signalling and disrupted mitochondrial substrate handling^41^.

While a substantial proportion of the metabolites and clinical biomarkers were found to mediate the effect of BMI clusters on T2D risk, no proteins reached statistical significance after correction for multiple testing. This likely reflects a combination of limited statistical power and biological differences between molecular layers. In particular, the number of available genetic instruments and eligible participants was more restricted for proteins than for metabolites, reducing our ability to detect significant mediation effects. Moreover, circulating proteins may represent more specific or individual biological processes, whereas metabolites are likely to capture downstream and integrative effects of multiple pathways influenced by adiposity. Consequently, metabolites may be more sensitive markers of the combined metabolic impact of BMI-related heterogeneity on T2D risk, while protein-mediated effects may be subtler and require larger sample sizes to be robustly detected. Among the proteins examined, adiponectin showed the strongest association. Consistent with its known role in enhancing glucose uptake, fatty acid oxidation, and insulin sensitivity, higher adiponectin levels in the protective cluster may contribute to its reduced T2D risk.

Based on gene mapping of SNPs within the clusters and subsequent enrichment analyses, the protective cluster was associated with significant downregulation and the risk clusters with upregulation of genes expressed in brain regions. This indicates that the risk-increasing clusters are more likely to influence BMI through food intake to a larger degree compared to the protective cluster. The high-risk clusters were predominantly enriched for biological processes related to vesicle-mediated transport, synaptic junction signalling, and adipocyte differentiation, whereas the protective cluster showed enrichment for pathways involved in nutrient response. This suggests that high risk might also be associated with disruptions in adipocyte differentiation and neuronal communication, whereas protective profiles are more linked to efficient nutrient and metabolic regulation. Enrichment analyses based on proteins implicated by the cluster-specific MR analyses revealed that the risk-increasing clusters were enriched for proteins involved in metabolic processes, particularly those related to small molecules, organic acids, and amino acids, as well as oxidoreductase activity. Many of these pathways are central to liver function and energy metabolism, consistent with enrichment for hepatocyte-related processes. Importantly, these metabolic and catabolic pathways are highly relevant to T2D and are consistent with the results observed for the metabolite mediators.

Obesity is often classified into metabolically healthy (MHO) and metabolically unhealthy subtypes. Yet this distinction is problematic, as individuals with MHO remain at elevated risk of T2D and other obesity-related conditions compared with normal-weight individuals^42^. Although genetic studies have identified variants that dissociate obesity from metabolic disease, they have not captured the broader heterogeneity of causal pathways contributing to obesity-related risk^19,20^. Most previous studies that have classified T2D into aetiological subgroups have used approaches different from ours, identifying categories related to adiposity, beta-cell dysfunction, insulin resistance, dyslipidaemia, or hepatic glucose handling. With new advances in technology and methodology, more granular classifications were achievable, involving use of imaging, genetics and other multi-omics^5,6,18–20,43,44^. For example, Coral et al^19^. identified genetic profiles linked to high obesity risk with either increased (concordant) or decreased (discordant) T2D risk with a more limited number of SNPs. Similar to our results, the discordant (protective) profile had a healthier cardiometabolic and metabolite profile, particularly for lipid fractions. Another study, by Martin et al. ^18^, performed a multivariate GWAS using body fat percentage (BFP) and metabolic biomarkers to identify two adiposity phenotypes. Thirty-eight unfavourable adiposity variants were associated with adverse metabolic profiles and higher risk of cardiometabolic diseases, while thirty-six favourable adiposity variants were linked to higher fat mass but healthier metabolic profiles and lower disease risk. Variants in the unfavourable group correlated with increased fat in the liver, visceral abdominal depots, and pancreas, whereas variants in the favourable group were associated with lower liver fat and protection from cardiometabolic diseases, including T2D. In a recent study, Abraham et al. ^21^ used MR-clust to investigate differential effects of adiposity (BMI and BFP) on T2D. They identified five BFP clusters (three risk-increasing, two protective) and three BMI clusters (two risk-increasing and one protective), and evaluated their effects on metabolites, anthropometrics, metabolic biomarkers, and inflammatory cytokines using cluster-specific PRSs. Unfavourable clusters were linked to adverse metabolic profiles and ectopic fat deposition in organs such as liver, pancreas, and VAT, while favourable clusters showed healthier metabolic profiles and lower disease risk. Imaging analyses revealed that all clusters were associated with increased subcutaneous fat, but protective clusters differed in muscle and organ volumes. In contrast to the previous studies, our study adds extensive omics profiling alongside clinical biomarkers and functional analyses.

While there are several strengths of our study, including the large sample size, the availability of both summary statistics and individual-level data, the use of causal inference by means of MR, and that we leveraged large-scale proteomics, metabolomics and other biomarkers data to enhance our understanding of molecular and biological pathways leading to T2D, there are also some weaknesses. We used data from participants of European ancestry, and the results may have limited generalisability in non-European populations and ethnicities. The “distinct” clusters identified the MR-clust algorithm are based on effect magnitude and therefore it is possible that observed clusters may represent differences in magnitude rather than distinct mechanistic pathways and may thus require more evaluation of their robustness. We also observed widespread horizontal pleiotropy in the MR analyses of the causal associations between NMR metabolites and T2D which potentially indicates the interconnectedness of pathways involved in energy metabolism. This phenomenon was also observed in the discovery study^32^ and therefore caution is urged in the interpretation of results for individual traits.

A further limitation of our study is that the identified clusters remain challenging to fully interpret mechanistically and clinically, particularly regarding distinguishing the two harmful clusters (C1 from C2). The variants contributing to all clusters span a spectrum of effects, from harmful to protective, and differences between clusters are best understood as gradients rather than strict qualitative distinctions. Some clusters, such as C4, do show a more uniquely different profile as compared to the two harmful clusters. However, individuals are likely to carry a mosaic of BMI increasing alleles from multiple clusters, adding an additional layer of complexity at the individual level. Derived from genetic variants, the clusters therefore represent a simplification of the complex and heterogeneous ways in which obesity influences T2D risk, yet they provide informative insights into the underlying biological mechanisms. Even as gradients, these clusters highlight meaningful variation and help clarify the diverse pathways linking obesity to T2D.

High-throughput technologies for targeted proteomics, metabolomics, and clinical biomarkers in large cohorts enabled this study. However, it should be noted that only a minor fraction of all human proteins and metabolites that are available in the human body were analysed in this study, and proteins were restricted to specific protein isoforms, and all measurements were from plasma. Also, while genetic instruments were identified for most metabolites and clinical biomarkers, only around half of proteins we analysed had genetic variants available for MR analyses. Therefore, our results should not be interpreted as representing all molecular pathways between obesity and T2D.

In summary, we have identified multiple molecular pathways linking obesity to T2D and demonstrated substantial heterogeneity in the causal effects of BMI, providing a more nuanced understanding of “protective” obesity variants and the MHO phenotype. Our findings suggest that two individuals with the same number of BMI-increasing alleles can have very different T2D risks, even at identical BMI. While our study focused on the differential effects of SNP clusters, and individuals carry unique combinations of these variants, complicating direct translation to single cases, the results illuminate molecular mechanisms through which different aspects of obesity influence T2D risk. These insights highlight potential targets for precision prevention strategies aimed at reducing disease burden.

## Methods

### Ethics

This study utilises individual-level data from the UK Biobank (UKB). All participants in the UKB provided written informed consent, and the study was conducted in compliance with the principles outlined in the Declaration of Helsinki. Ethical approval for the UK Biobank project was granted by the National Research Ethics Committee (11/NW/0382), allowing bona fide researchers to conduct research on UK Biobank data without separate ethical approval. Approval for the use of data from UK Biobank for this study was granted by the UK Biobank Access Committee (application number: 15152). Ethical approval to store and analyse UK Biobank data in Sweden was also granted by the Swedish Ethical Review Authority (dnr: 2020–04415 and 2024-07639-02).

### GWAS data for BMI and T2D and meta-analyses for clustering

For the initial clustering, we specifically selected GWAS summary statistics from cohorts that excluded participants from the UKB to enable validation and further evaluation of the clusters in an independent sample/cohort. We obtained summary statistics data for BMI from the Genetic Investigation of ANthropometric Traits (GIANT) consortium^45^ and FinnGen data release 9 (R9)^46^. The GIANT consortium meta-analysis included 339,224 participants (of which 322,154 were of European ancestry and included in our analyses) from 125 studies. The FinnGen study is a nationwide effort that combines data from multiple biobanks in Finland and the R9 data release consisted of 377,277 individuals. We combined the GIANT and FinnGen data via fixed-effect, inverse-variance weighted meta-analysis which resulted in 15,254 genome-wide significant (two-sided *P* < 5×10^−8^) SNPs. From these, we selected 513 independent BMI SNPs by clumping (with PLINK v1.9)^47^ with a 500 kb window and r^2^ = 0.001, to be used in downstream analyses.

GWAS summary statistics data for T2D were obtained from the DIAGRAM (DIAbetes Genetics Replication And Meta-analysis) consortium which involved 74,124 cases and 824,006 controls of European ancestry from 32 studies^48^. From these data, we extracted summary statistics information for the 513 independent BMI SNPs identified in the previous step. We performed quality control (QC) using the TwoSample MR package^49^ to exclude non-biallelic or non-inferable palindromic SNPs, and harmonised the two datasets so that the effect on the two phenotypes was estimated for the same allele. We set automated proxy search and replacement at an r^2^ => 0.8 threshold for variants that were filtered out during the QC and harmonisation step. After this step, 463 independent SNPs remained for further analyses using MR-clust. For all MR analyses and other statistical tests, R v4.1.1 was used^50^.

### Identifying clusters of causal heterogeneity

To identify distinct clusters of SNPs, potentially representing different biological pathways between obesity and T2D, we used the MR-clust algorithm that utilises GWAS summary statistics to classify SNPs based on their effect on BMI and on T2D risk, and is implemented in the MR-clust R package^28^. Besides classifying SNPs, the algorithm also quantifies an average effect for each cluster referred to as the cluster mean. In addition, for each cluster, we performed conventional two-sample IVW MR analysis that enabled us to compare the cluster means derived from MR-clust to the IVW causal estimate. For all IVW estimates, the multiplicative random-effects model was used throughout the study and two-sided *P*-values are reported.

### The MR-clust algorithm

In MR, genetic variants are used as instrumental variables (IV) for an exposure, to estimate the unconfounded effect of the exposure on an outcome. The underlying expectation is that the ratio between the effect of each IV on the outcome and the exposure, i.e., the causal estimates should be similar. However, MR analyses commonly suffer from heterogeneity, which can be solved by simply removing heterogenous variants or modifying variance weights^51^. This procedure, however, does not account for the possibility that the heterogeneity reflects separate groups (clusters) of genetic variants with distinct causal effects. In this study, the underlying hypothesis is that genetic variants yielding similar MR estimates of the effect of a risk factor on an outcome may reflect shared biological pathways and hence cluster together^28^, whereas heterogeneity suggests the presence of distinct biological pathways with cluster-specific effects.

In line with this, for a set of genetic variants, the MR-clust algorithm identifies ***K*** substantive clusters, with ***K*** being optimised by the algorithm. The algorithm further allows for identification of two disjoint clusters, i.e., a null and a junk cluster. The null cluster represents variants that do not suggest a causal effect of the risk factor on the outcome while the junk cluster represents variants that are not classifiable into any other cluster. The inclusion of these two cluster types in the modelling reduces the risk of detecting spurious clusters. The algorithm adopts a mixture model where the ratio estimate of each genetic variant is assumed to follow a normal distribution if the variant is a member of a substantive cluster or the null cluster, or a generalised t-distribution if it is a member of the junk cluster. For a given number of substantive clusters, the cluster means (*θ*_*k*_) and the mixture proportions (π_*k*_) of the mixture model are estimated using an expectation-maximisation algorithm. Finally, the number of substantive clusters is determined by minimising the Bayesian information criterion, selecting the best mixture model^28^. SNPs were assigned to the cluster with the highest MR-clust membership probability, without applying a strict probability threshold. This allowed for more variants to be assigned to each cluster and to be included in downstream analyses, compared to if a stringent probability threshold had been used. In our analyses, the lowest membership probability for any cluster was 48.6%. We compared this to an 80% probability threshold, which resulted in very few SNPs being assigned to each cluster (Supplementary Table 1). Such a threshold would have decreased the power in downstream analyses.

### Annotation of SNPs to genes and enrichment analyses

To annotate SNPs to their respective genes, we used FUMA^29^ (functional mapping and annotation of genetic associations) with the 463 SNPs as input. First, the SNP2GENE function was applied to annotate the SNPs to genes. Second, these genes were used as input to the GENE2FUNC function to perform differential expression of genes. We further used g:Profiler for pathways analyses using various databases including, for example, KEGG; Reactome and WikiPathways and Gene Ontology (GO) terms (molecular functions, biological processes and cellular components)^30^.

### UK Biobank (UKB) individual-level data

For analyses that required individual-level data, we used the UKB, which is a cohort of more than 500,000 participants (aged between 37-73 years at enrolment), enroled across 22 study centres in the UK. At recruitment, between 2006 and 2010, the participants’ anthropometric measurements were taken, and they answered survey questions related to lifestyle and other health determinants. Blood, urine and saliva samples were obtained for biochemical measures and stored for future analyses

Genotyping in the UK Biobank was performed using two arrays (UK BiLEVE Axiom array − 49,950 participants; and UKB Axiom Array − 438,427 participants) in sequential batches, and subsequent imputations of unmeasured genotypes. Details about the cohort, samples and data processing are described in Bycroft et al. ^52^.

Proteomic data were available for a subset of individuals (*N* = 54,219) selected for plasma protein assaying in the UKB-PPP (UKB Pharma Proteomics Project) which is a collaboration between the UKB and 13 pharmaceutical companies. Briefly, plasma protein levels were measured using the OLINK Explore 3072 platform where a total of 2923 unique proteins were measured (normalised protein expression, NPX) across eight protein panels followed by quality control (QC) and batch normalisation^31^.

BMI was estimated at baseline an extracted from field 23104, or where values were missing, BMI was calculated from height (field 50) and weight (field 21002), where available. Age and sex as well as self-reported smoking and alcohol consumption were retrieved for the initial enrolment visit (the same time as BMI was extracted and when blood samples for proteomic analyses were taken). T2D incidence was defined as the date of first reported non-insulin-dependent diabetes mellitus (Field ID 130708). This variable combines information from multiple sources, including self-reports and national registers, with most cases based on hospital admissions, indicating the first diagnosis of T2D. Incidence of coronary artery disease (CAD), stroke, and chronic kidney disease (CKD) were identified using Field IDs: CAD (42,000, 42,002, and 42,004), ischaemic stroke (42,008) and CKD (132,032).

For analyses requiring age at onset, the date was converted to age by subtracting the participant’s birth date. Participants without a recorded date of first T2D diagnosis were considered non-cases. Other terms like age-squared and interaction terms for sex-age and sex-age-squared were derived from the above listed variables. Other technical variables included were participation in the PPP cohort, protein assay batch number, time to processing (number of days from sample collection to processing for proteomic assaying), genetic array and the first 20 genetic principal components (PCs). See Supplementary Table 4 for information on UKB variables.

We included unrelated individuals of European ancestry, and after excluding those with missing BMI measurements (due to unavailable height or weight data), the final sample comprised 360,813 participants, of whom 38,562 had protein measurements. One protein was excluded because more than 90% of its measurements were missing, leaving 2922 unique proteins for downstream analyses.

### Cluster-specific BMI polygenic risk scores (PRS) and T2D in UK Biobank

To assess the effect of the different BMI clusters on T2D in UKB, we generated cluster-specific BMI PRSs. These PRSs were constructed using the SNPs assigned to each cluster, with SNP weights corresponding to effect sizes from the FinnGen+GIANT meta-analysis. Each UKB participant was scored using PLINK v1.9. We then used adjusted Cox proportional hazards regression, with age (in years) as the primary time scale, to estimate the association between T2D and each of the four cluster-specific PRSs. In addition, we plotted the unadjusted cumulative incidence of T2D for individuals in the top and bottom 50% of each cluster-specific PRS distribution and used the log-rank test to check whether there was a significant difference between the cumulative incidences.

### GWAS summary statistics for molecular traits as outcomes in MR

To assess the effects of the cluster-specific BMI instruments on molecular traits, we extracted the effect of the BMI instruments on various molecular traits. For proteins, we used individual-level data from the UKB as described in a previous paragraph, which also enabled sex-stratified analyses. We performed linear regression with normalised protein expression levels as the outcome, adjusting for age, age squared, sex, and 20 principal components, to estimate the effects of the cluster-specific BMI instruments on proteins, using PLINK v1.9.

We also assessed the causal effect of the cluster specific BMI instruments on NMR metabolites and on various clinical biomarkers: blood and urine biomarkers, including glycaemic biomarkers (fasting and random glucose, HbA1c, insulin sensitivity index, proinsulin, fasting insulin, two-hour glucose, and insulin fold-change). Here we used most recent GWAS summary data for each respective trait. Data for clinical biomarkers were extracted from a UKB GWAS of blood and urine tests from 363,228 unrelated participants of predominantly European ancestry (n = 318,953 white British)^33^. Data for NMR metabolites were obtained from a GWAS combining the UK and Estonian biobanks for a total of 619,372 individuals^32^. Data for glycaemic biomarkers were extracted for each respective study from the Meta-Analyses of Glucose and Insulin-related traits Consortium, MAGIC^34–36^.

### Two-sample MR to estimate the effect of BMI-clusters on molecular traits and between-cluster heterogeneity

We used a two-sample MR design, using cluster-specific BMI SNPs as instruments for each cluster. To minimise sample overlap in the two-sample MR, we used the effect estimates of the instruments on BMI from the FinnGen+GIANT meta-analyses and extracted the effect estimates on the molecular traits, mainly from UKB (See previous paragraph). To compute the MR estimates, we used the IVW and MR-Egger methods implemented in the TwoSampleMR package. The multiplicative random-effects IVW model was considered the primary analysis, while MR-Egger was used as a sensitivity analysis to test for directional pleiotropy.

The molecular traits were intended to provide further information on the different molecular pathways separating the four BMI clusters. For such traits, we assumed that the IVW MR estimates differ between clusters. To assess whether there is such heterogeneity in cluster effects for a given biomarker, we applied a chi-square test that compares the IVW MR estimates, accounting for the uncertainty of each cluster estimate (assuming the standard errors are known). At this stage, we only included molecular traits that passed the threshold for significance described above. For each individual biomarker, the heterogeneity statistic, *T*, is given by Eq. 1:

1 $${{{\rm{T}}}}={\sum \left(\hat{{{\beta }}}_{{{\rm{i}}}}-\bar{{{\beta }}}\right)}^{2}/{{{{\rm{SE}}}}_{{{\rm{i}}}}}^{2}$$

where *β̂ᵢ* and *SEi* are the IVW MR effect estimate and standard error for cluster *i* = 1,…, 4, respectively, while *β̄* denotes the weighted mean effect across the four clusters. Under the null hypothesis of no difference in effect, the statistic *T* is chi-square distributed with three degrees of freedom (equal to the number of clusters minus one). A significant p-value suggests that the cluster effects differ more than expected by chance, capturing both general heterogeneity and monotonic trends.

In both the MR and the heterogeneity test we applied FDR correction using the Benjamini–Hochberg procedure separately for each omics data type (proteins, NMR metabolites, and clinical biomarkers) but across all clusters. Associations with an FDR-adjusted *P* < 0.05 were considered statistically significant.

### Enrichment analyses for cluster-specific proteins

Enrichment analyses were conducted to identify enriched terms among the cluster-specific proteins, using the web-based version of g:Profiler (https://biit.cs.ut.ee/gprofiler)^30^. The analyses included proteins that (i) were significantly affected by at least one cluster in the cluster-specific BMI to protein IVW MR analyses, and (ii) showed a significant heterogeneity in IVW estimates between clusters. The total list of proteins was used as the background set. Enrichment was evaluated across multiple annotation sources, including GO terms (Molecular Function, Cellular Component, Biological Process), biological pathways (KEGG, Reactome, WikiPathways), and protein databases (Human Protein Atlas). Multiple testing correction was applied using the g: SCS algorithm, which computes experiment-wide adjusted two-sided *P*-values for GO and pathway enrichment analyses. This approach corresponds to a significance threshold of α = 0.05, ensuring that at least 95% of matches above the threshold are statistically significant, and is more conservative than an FDR adjustment.

### Causal effect of molecular traits on T2D

Molecular traits that were significant in the cluster-specific MR analyses for any of the clusters, and for which significant between-cluster heterogeneity was detected, were carried forward to assess their causal effect on T2D using two-sample MR analyses based on summary-level GWAS data. The same data sources described above were used for the molecular data. However, in this step, instruments to be used in the MR analyses were identified for each of the molecular trait as the independent lead-SNPs from the previous GWASes. For proteins, we used data from Sun et al^31^, from which the pre-identified lead SNPs were selected as instruments (two-sided *P*-value < 5 × 10⁻⁸), and their respective effect sizes on protein levels were extracted. For proteins, we aimed to use SNPs influencing the expression of each significant protein (i.e., pQTLs) as instruments for the respective protein. Because pQTLs are often pleiotropic (i.e., some trans-regulatory SNPs are associated with multiple proteins), we included only proteins with at least three significant SNPs, of which at least one was a *Cis*-regulatory SNP, to enable the evaluation of heterogeneity and pleiotropy in the MR analyses.

For the NMR metabolites we used the pre-defined list of independent lead SNPs for each metabolite trait which was defined by *P*-value < 5 × 10^−8^ as provided by Tambets et al^32^. For Clinical biomarkers (including specific glycaemic biomarkers) we used the full summary statistics (as described above) to identify independent significant SNPs to be used as instruments for the exposure in the MR analysis, using a threshold of two-sided *P*-value < 5×10^−8^ and clumping r^2^ = 0.001 and 500 kb window.

Effect estimates for these instruments on T2D were extracted from GWAS summary statistics data from the DIAGRAM consortium^53^, the same data as used in the clustering (see above). We also performed QC and harmonisation for each omics-T2D (exposure and outcome) sets as described above. To compute the MR estimates we used the multiplicative random effects IVW (primary results) and MR-Egger (sensitivity analyses), and Cochran’s Q statistic was used to estimate a *P*-value for heterogeneity. The analyses were carried out using the TwoSampleMR package in R statistical software v. 4.1.1^49^. A within omics-type FDR-adjusted two-sided *P* < 0.05 was considered statistically significant.

### Mediation analyses

To estimate to what degree the molecular traits mediate the effect of obesity on T2D, we performed formal mediation analyses on molecular traits that showed significant associations in all preceding MR steps: (i) BMI to biomarker (IVW MR), (ii) heterogeneity in IVW estimate between clusters (chi-square test), and (iii) biomarker to T2D (IVW MR), with a step-wise empirical FDR-adjusted *P* < 0.05 in each step. Mediation analyses were conducted only for the two risk-increasing clusters (C1 and C2) and the protective cluster (C4). The Null cluster (C3) was excluded because it did not show a significant total effect. The mediation analysis was performed using the product-of-coefficient method for each biomarker per BMI cluster^54^. The indirect (mediated) effects were estimated by taking the product of the effect of BMI on biomarker and the effect of the biomarker on T2D, with the effect being the IVW estimates in the MR analyses described above. We computed the percentage mediated as the ratio between indirect and total effect, where the total effect is the IVW estimate from the BMI (FinnGen+GIANT Meta-analyses) to T2D (DIAGRAM) MR analyses. The Sobel test was used to assess whether a biomarker significantly mediates the causal effect of BMI on T2D. Using the product-of-coefficients approach, the Sobel test statistic (*Z*) was calculated with a normal approximation as in Eq. 2:

2 $$Z=\frac{{ab}}{\sqrt{{b}^{2}\,S{E}_{a}^{2}+{a}^{2}\,S{E}_{b}^{2}}}$$

where *a* is the causal effect of BMI on biomarker and *b* is the causal effect of the biomarker on T2D, and *SE*_*a*_ and *SE*_*b*_ are the standard errors for respective estimates. The corresponding two-sided *P*-value was obtained assuming that *Z* is distributed according to a standard normal under the null.

### Two-sample MR to estimate the effect of BMI-clusters on imaging-derived and fat distribution traits as well as cardiometabolic diseases

MRI of participants is ongoing in UK Biobank, with a target of 100,000 individuals undergoing imaging, and repeat imaging planned for 70,000 participants. At the time of study initiation (22 May 2023), raw imaging data from approximately 40,000 participants were available from the initial imaging phase, which began in 2014. We utilised quantitative body composition measures derived from the raw imaging data and provided by UK Biobank, including abdominal body composition (VAT volume; data fields 21085–21086), liver volume and fat content (data fields 21088–21089), and pancreatic volume and fat content (data fields 21087 and 21090). In addition, WHR had been calculated from anthropometric measurements obtained at the same time-point as the imaging. Alongside T2D, we also evaluated the cluster specific effects on four additional cardiometabolic outcomes: myocardial infarction from the CARDIoGRAM consortium^55^, cerebral infarction^56^ from the MEGASTROKE consortium and essential hypertension and hyperlipidaemia from FinnGen^46^. We extracted the effect estimates for the cluster specific IVs from summary statistics for both imaging-derived traits and WHR as well as for the cardiometabolic diseases.

### Reporting summary

Further information on research design is available in the Nature Portfolio Reporting Summary linked to this article.

## Supplementary information

Supplemenary Information

Description of Additional Supplementary Files

Supplementary Data

Reporting Summary

Transparent Peer Review file

## Source data

Source data

## Data Availability

The UKB data used in this study are subject to controlled access due to the inclusion of sensitive participant information. Data are available to bona fide researchers upon application to the UKB via the Access Management System [https://www.ukbiobank.ac.uk]. Access is granted following review and approval of a research proposal and is subject to a data use agreement that restricts data use to the approved project and prohibits re-identification of participants. The UKB typically responds to access requests within a defined review period as outlined on their website. BMI summary statistics data are from FinnGen [https://www.finngen.fi/en/access_results], file name summary_stats_finngen_R9_BMI_IRN.gz, and GIANT, file name SNP_gwas_mc_merge_nogc.tbl.uniq.gz. T2D data were downloaded from DIAGRAM using the file: Mahajan.NatGenet2018b.T2D-noUKBB.European.txt. The pQTL data were obtained from the discovery credible sets in the UKB as reported in Sun B. et al. (ST16)^31^. Full summary statistics for the GWAS of NMR metabolites are available from the GWAS catalogue [https://www.ebi.ac.uk/gwas/home], under accession numbers GCST90449363 - GCST90451603. Significant lead SNPs are available on Zenodo [10.5281/zenodo.13937265]. Data for clinical biomarkers were obtained from Sinnott-Armstrong et al^33^. [10.35092/yhjc.12355382] for the 35 biomarkers available in the UKB, and from the MAGIC consortium [https://magicinvestigators.org] for glycaemic traits. GWAS summary statistics for imaging-derived traits and WHR are available at Zenodo [10.5281/zenodo.19590199]. Source data are provided with this paper.

## Code availability

The code used in the analyses in this study is available on Zenodo [10.5281/zenodo.19422005].
