# Supplementary material for: Genetic and molecular signatures highlight diverse pathways linking obesity to type 2 diabetes
Source: Nat Commun. 2026 Jul 8;17:5680. doi: 10.1038/s41467-026-74675-9 (PMC13347065; doi:10.1038/s41467-026-74675-9)
Supplement: Supplementary file 1 — Supplemenary Information [file 41467_2026_74675_MOESM1_ESM.pdf]

SUPPLEMENTARY INFORMATION

Genetic and molecular signatures highlight diverse pathways linking obesity to type 2 diabetes

Table of Contents

Supplementary Table 1. Number of SNPs assigned to different clusters depending on the cluster probability cut-off used. 2

Supplementary Table 2. UKB Participants' baseline characteristics 3

Supplementary Table 3. Mendelian randomization (MR) results for the effect of each BMI cluster on clinical biomarkers, restricted to significant findings only. 4

Supplementary Table 4. Field codes of variables used 5

Supplementary Figure 1. Differentially expressed genes associated with the four clusters. 6

Supplementary Figure 2. Distributions of the cluster-specific BMI PRSs in the UKB. 7

Supplementary Figure 3. Cluster-specific MR estimates for the effect of BMI on protein levels in sex-stratified analyses. 8

Supplementary Figure 4. NMR metabolites with decreased T2D risk and their cluster-specific association with BMI disaggregated by metabolite group 9

Supplementary Figure 5. NMR metabolites with increased T2D risk and their cluster-specific association with BMI disaggregated by metabolite group 10

Supplementary Figure 6. Cluster-specific associations between BMI and imaging-derived traits 11

Supplementary Table 1. Number of SNPs assigned to different clusters depending on the cluster probability cut-off used.

Cluster mean MR estimates were calculated by MR-Clust as inverse-variance weighted means of SNP-specific MR effects within each cluster, and 95% confidence intervals (CIs) were derived from the corresponding standard errors. Summary statistics for BMI were obtained from the GIANT consortium (N = 322,154 participants) and FinnGen (data release 9; N = 377,277 participants). T2D data were obtained from the DIAGRAM consortium (N = 74,124 cases and N = 824,006 controls). Odds ratios (ORs) correspond to the exponentiated cluster mean estimates. All statistical tests were two-sided.

Cluster	OR	95% CI	P-value	N SNPs	Probability cut-off for SNPs to be assigned to a cluster
C3	0.700	0.606-0.807	9.8E-07	56	50%
C4	0.075	0.052-0.109	4.5E-42	11	50%
C2	2.345	2.206-2.492	1.2E-165	307	50%
C1	6.168	5.583-6.815	7.4E-280	85	50%
C3	0.613	0.494-0.760	8.1E-06	19	80%
C4	0.075	0.052-0.109	4.5E-42	11	80%
C2	2.275	2.008-2.578	4.0E-38	24	80%
C1	6.492	5.085-8.287	5.9E-51	22	80%

Supplementary Table 2. UKB Participants' baseline characteristics

Attribute	*Females*	*Males*
N (%)	194184 (53.8%)	166587
BMI (mean, SD)	27.04 (5.14)	27.85 (4.23)
Age (mean, SD)	56.68 (7.9)	57.13 (8.08)
Alcohol: Never	8368	2790
Previous	6927	5297
Current	178712	158360
Smoking: Never	115121	81400
Previous	61828	65014
Current	16587	19587
T2D: No	182033	148001
Yes	12151	18586
CAD: No	188383	150856
Yes	5801	15731
CKD: No	183264	155562
Yes	10920	11025
Stroke: No	190768	161157
Yes	3416	5430

Supplementary Table 3. Mendelian randomization (MR) results for the effect of each BMI cluster on clinical biomarkers, restricted to significant findings only.

The table shows all clinical biomarkers that were significantly affected by at least one BMI cluster, displayed significant heterogeneity between clusters, and were also significant in the subsequent clinical biomarker → type 2 diabetes (T2D) MR analyses after false discovery rate (FDR) adjustment. MR estimates were obtained using inverse-variance weighted (IVW) analyses, and heterogeneity between clusters was assessed using a chi-square test. All statistical tests were two-sided, and p-values were adjusted for multiple comparisons using the FDR method. Most traits were estimated in unrelated participants self-reported as White British (N = 318,953), except for three traits (2-hour glucose, fasting glucose, and fasting insulin), which were measured in the MAGIC consortium (N = 476,326 for random glucose; N = 281,416 for 2-hour glucose, fasting glucose, HbA1c, and fasting insulin). Estimates highlighted in green represent favourable biomarkers, associated with a lower risk of T2D: positively associated with the protective cluster (C4) and negatively associated with the harmful clusters (C1 and C2). Estimates highlighted in red represent non-favourable biomarkers, associated with increased risk of T2D: negatively associated with the protective cluster and positively associated with the harmful clusters. Estimates highlighted in blue denote biomarkers where the protective and harmful clusters do not show opposing effects, but the protective cluster shows a large effect. These patterns are consistent with potential protective or harmful effects mediated by the respective BMI clusters.

			MR results IVW Cluster-specific BMI-> Clinical biomarkers								***Clinical biomarkers -> T2D***		
			C1(High-risk)		*C2 (Medium-risk)*		*C3 (Null)*		*C4 (Protective)*		*Inverse variance weighted*		
Clinical biomarkers	***Clinical biomarkers information***	***Cohort***	*Estimate*	*SE*	*Estimate*	*SE*	*Estimate*	*SE*	*Estimate*	*SE*	*Estimate*	*SE*	*p-value*
AST_ALT_ratio	*Ratio of AST (aspartate aminotransferase) and ALT (alanine aminotransferase)*	*UK Biobank*	*-0.252*	*0.031*	*-0.202*	*0.018*	*-0.056*	*0.051*	*0.511*	*0.172*	***-0.579***	*0.082*	*1.21E-12*
SHBG	*Sex Hormone-Binding Globulin – a protein that binds sex hormones in the blood*	*UK Biobank*	*-0.310*	*0.028*	*-0.237*	*0.019*	*-0.023*	*0.056*	*1.102*	*0.296*	***-0.302***	*0.058*	*1.69E-07*
HDL_cholesterol	*High-Density Lipoprotein cholesterol*	*UK Biobank*	*-0.312*	*0.039*	*-0.235*	*0.020*	*-0.163*	*0.069*	*0.386*	*0.109*	***-0.280***	*0.040*	*1.47E-12*
Apolipoprotein_A	*Main protein in HDL particles*	*UK Biobank*	*-0.249*	*0.040*	*-0.195*	*0.019*	*-0.185*	*0.060*	*0.214*	*0.107*	***-0.243***	*0.039*	*3.43E-10*
Urate	*Uric acid – a waste product of purine metabolism*	*UK Biobank*	*0.309*	*0.035*	*0.231*	*0.023*	*0.049*	*0.067*	*-0.263*	*0.214*	***0.121***	*0.043*	*0.00522*
Gamma_glutamyltransferase	*GGT – liver enzyme commonly used to evaluate liver and bile duct function*	*UK Biobank*	*0.218*	*0.037*	*0.143*	*0.019*	*-0.009*	*0.055*	*-0.368*	*0.230*	***0.129***	*0.036*	*0.000361*
IGF_1	*Insulin-like Growth Factor 1*	*UK Biobank*	*-0.073*	*0.048*	*-0.051*	*0.026*	*-0.126*	*0.053*	*-0.971*	*0.326*	***0.133***	*0.037*	*0.000282*
Triglycerides	*Fat molecules in the blood*	*UK Biobank*	*0.155*	*0.048*	*0.155*	*0.018*	*-0.012*	*0.044*	*-0.461*	*0.144*	***0.210***	*0.050*	*3.19E-05*
Alanine_aminotransferase	*ALT – liver enzyme; high levels indicate liver damage*	*UK Biobank*	*0.288*	*0.035*	*0.188*	*0.016*	*0.084*	*0.046*	*-0.451*	*0.226*	***0.513***	*0.064*	*8.24E-16*
Sodium_in_urine	*Sodium in urine – used to assess fluid and electrolyte balance*	*UK Biobank*	*0.140*	*0.030*	*0.109*	*0.015*	*0.070*	*0.040*	*-0.104*	*0.078*	***0.631***	*0.300*	*0.035387*
*Hba1c_UKB **	*HbA1c – long-term measure of blood sugar control over 2–3 months*	*UK Biobank*	*0.320*	*0.029*	*0.184*	*0.017*	*-0.094*	*0.044*	*-0.939*	*0.206*	***0.721***	*0.058*	*1.9E-35*
Two_hr_glu	*Blood glucose 2 hours after glucose load – used in oral glucose tolerance test*	*MAGIC*	*0.027*	*0.054*	*-0.105*	*0.031*	*-0.578*	*0.127*	*-0.599*	*0.355*	***0.829***	*0.357*	*0.020242*
Glucose_UKB	*Blood glucose – current level of sugar in the blood*	*UK Biobank*	*0.216*	*0.020*	*0.119*	*0.015*	*-0.017*	*0.043*	*-0.306*	*0.146*	***1.191***	*0.134*	*4.8E-19*
*Hba1c_MAGIC**	*HbA1c – long-term measure of blood sugar control over 2–3 months*	*MAGIC*	*0.065*	*0.009*	*0.027*	*0.006*	*-0.024*	*0.016*	*-0.121*	*0.070*	***1.283***	*0.345*	*0.000199*
Fasting_ins	*Fasting insulin – insulin level after fasting, used to assess insulin resistance*	*MAGIC*	*-0.007*	*0.018*	*-0.025*	*0.009*	*-0.113*	*0.025*	*-0.241*	*0.150*	***1.475***	*0.693*	*0.03342*
Fasting_glu	*Fasting glucose – blood sugar level after fasting*	*MAGIC*	*-0.010*	*0.015*	*-0.012*	*0.007*	*-0.125*	*0.024*	*-0.353*	*0.084*	***1.738***	*0.266*	*6.57E-11*
Random_glucose	*Random blood glucose – measures blood sugar without fasting*	*MAGIC*	*0.016*	*0.002*	*0.008*	*0.001*	*-0.004*	*0.005*	*-0.032*	*0.012*	***9.260***	*1.271*	*3.27E-13*

Supplementary Table 4. Field codes of variables used

Phenotype/ Variable	***UKB Field code***
Age	*21022*
Sex	*30*
BMI	*23104*
Smoking status	*20116*
Alcohol consumption	*20117*
Genetic Batch	*22000*
UKB centre	*54*
Type-2 diabetes	*130708*
CKD	*132032*
CAD	*42000, 42002 and 42004*
Ischemic stroke	*42008*
Height	*50*
Weight	*21002*
VAT volume	*21085 to 21086*
Liver volume and fat content	*21088 to 21089*
Pancreatic volume and fat content	*21087 and 21090*
Genetic principal components	*22009*
Proteomic data	
Participation in the PPP cohort	*30903*
Time to processing for proteins	*Time to processing: Calculated as difference between Olink processing start date and date of sample collection. Information provided in "resources" tab of Protein Biomarkers (https://biobank.ndph.ox.ac.uk/showcase/label.cgi?id=1839)*
Protein assay batch number	*30901*

CKD = chronic kidney disease; CAD = coronary artery disease, VAT = visceral adipose tissue

a) Cluster 1- High-risk

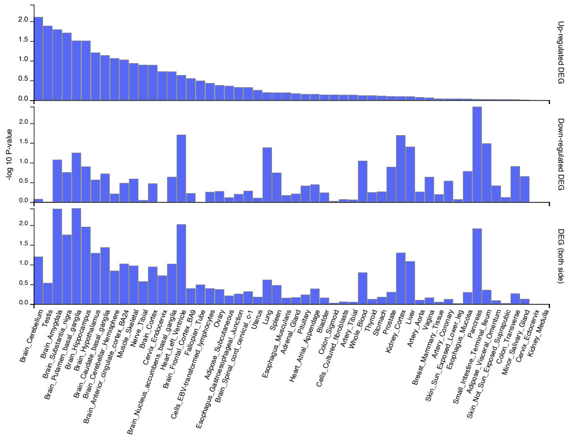

b) Cluster 2 - Medium-risk

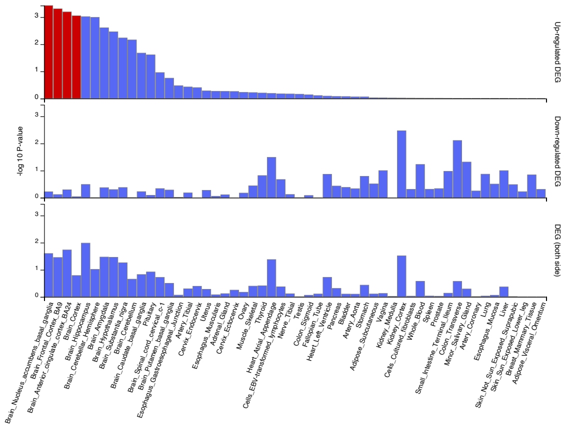

c) Cluster 3 - Null

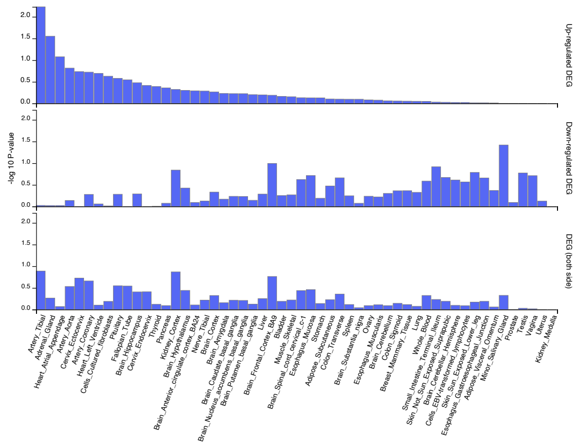

d) Cluster 4 – Protective

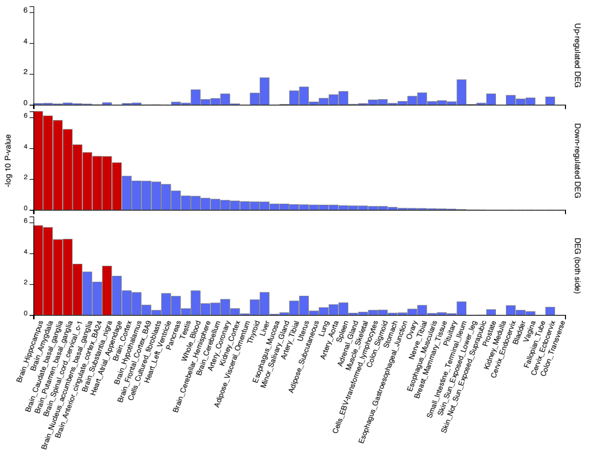

Supplementary Figure 1. Differentially expressed genes associated with the four clusters. *SNPs for each cluster (Supplementary Data 1) were annotated to genes using the SNP2GENE function in FUMA. Functional enrichment analyses were then performed using the GENE2FUNC function, including assessment of differential gene expression across 54 tissues based on GTex v8. Enrichment analysis was conducted using the hypergeometric test as implemented in g:Profiler. Padj - the experiment-wide adjusted Red bars indicate significant enrichment at Bonferroni corrected two-sided P-value of ≤ 0.05.*

.

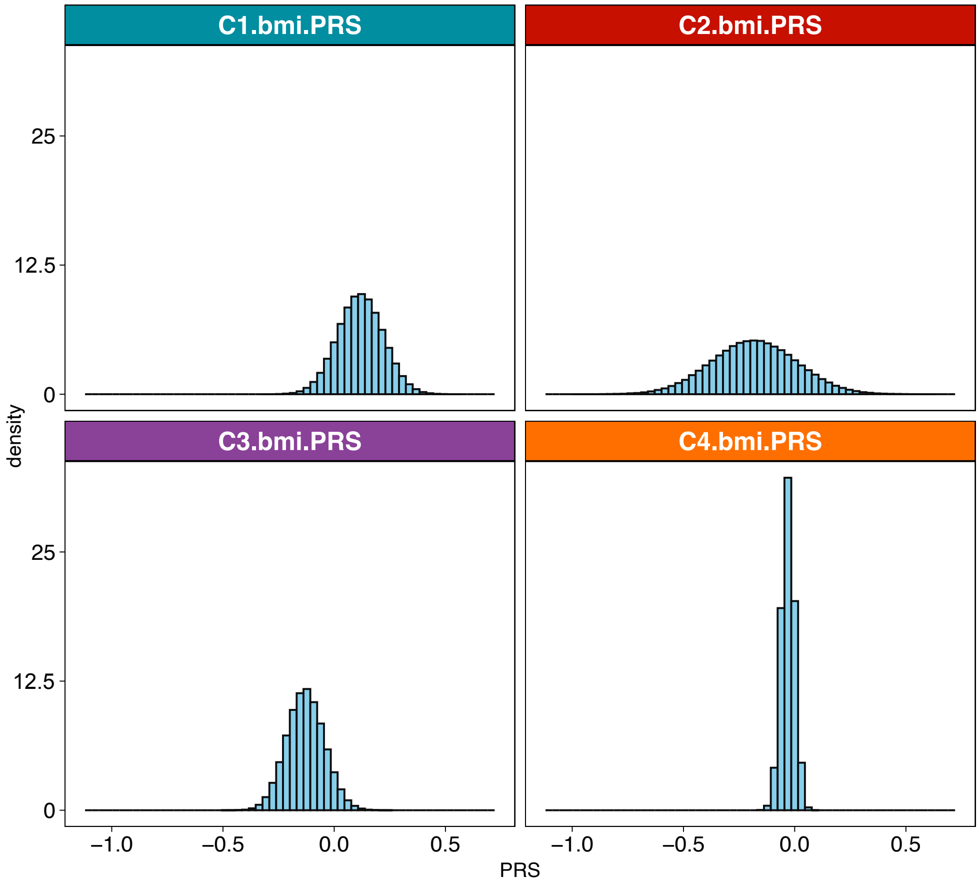

Supplementary Figure 2. Distributions of the cluster-specific BMI PRSs in the UKB.

The x-axis shows the PRS in standard deviation (SD) units for the four clusters, and the y-axis shows the percentage of individuals within each PRS interval. The total sample size is 360,771 UK Biobank participants.

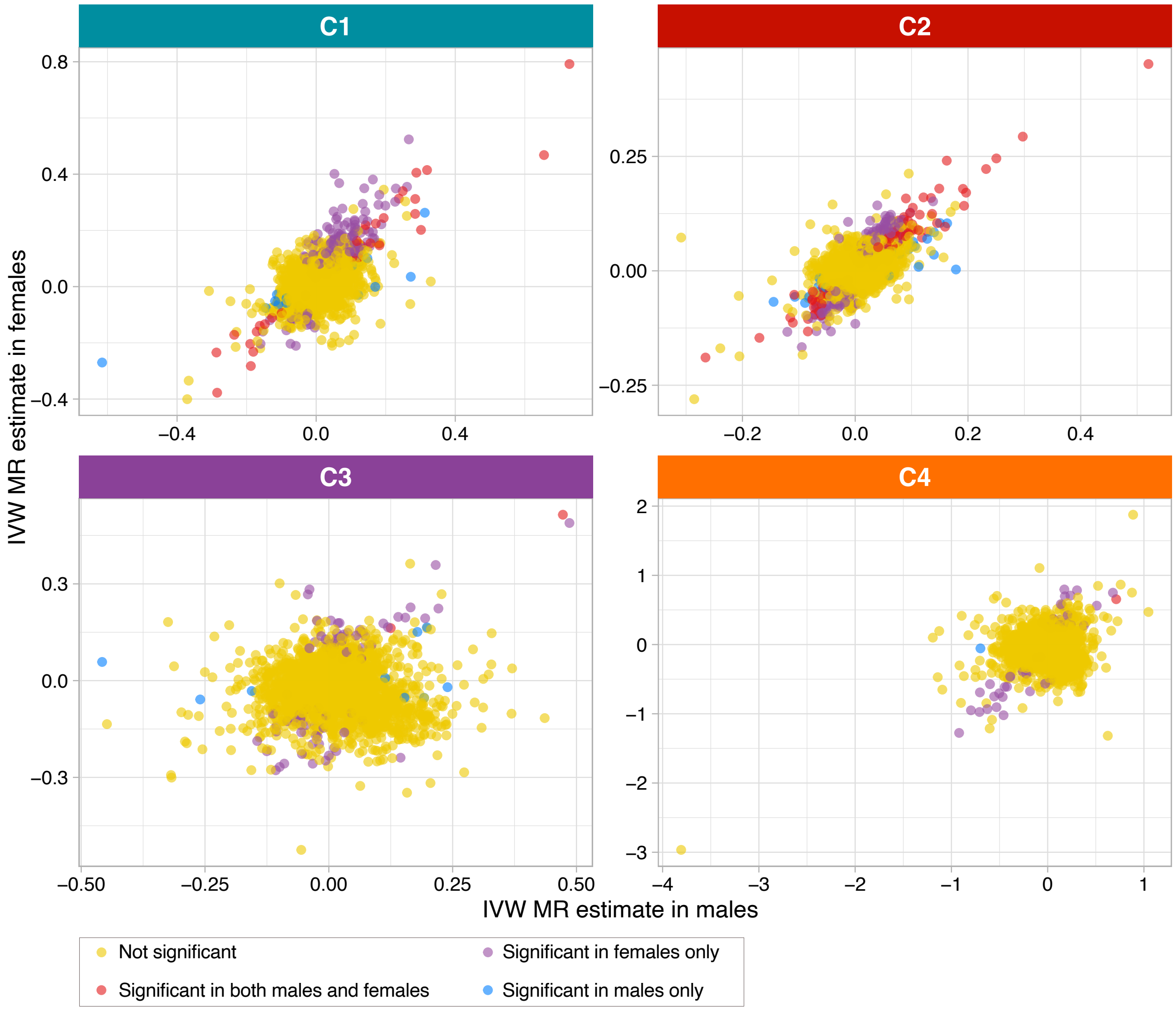

Supplementary Figure 3. Cluster-specific MR estimates for the effect of BMI on protein levels in sex-stratified analyses.

The four plots show the IVW MR estimates for each protein across the four clusters. Each point in the plots represents one of the 2,918 proteins, with the IVW MR estimate in males on the X-axis and in females on the Y-axis. Protein measures obtained from a total of 54,219 UKB participants were included in the analysis of which 53.8% are females. Points are color-coded to indicate the significance of the association with a significance threshold of FDR < 0.05. Yellow- not significant in any of the sexes, red – significant in both sexes, blue – significant in males only, and lilac – significant in females only.

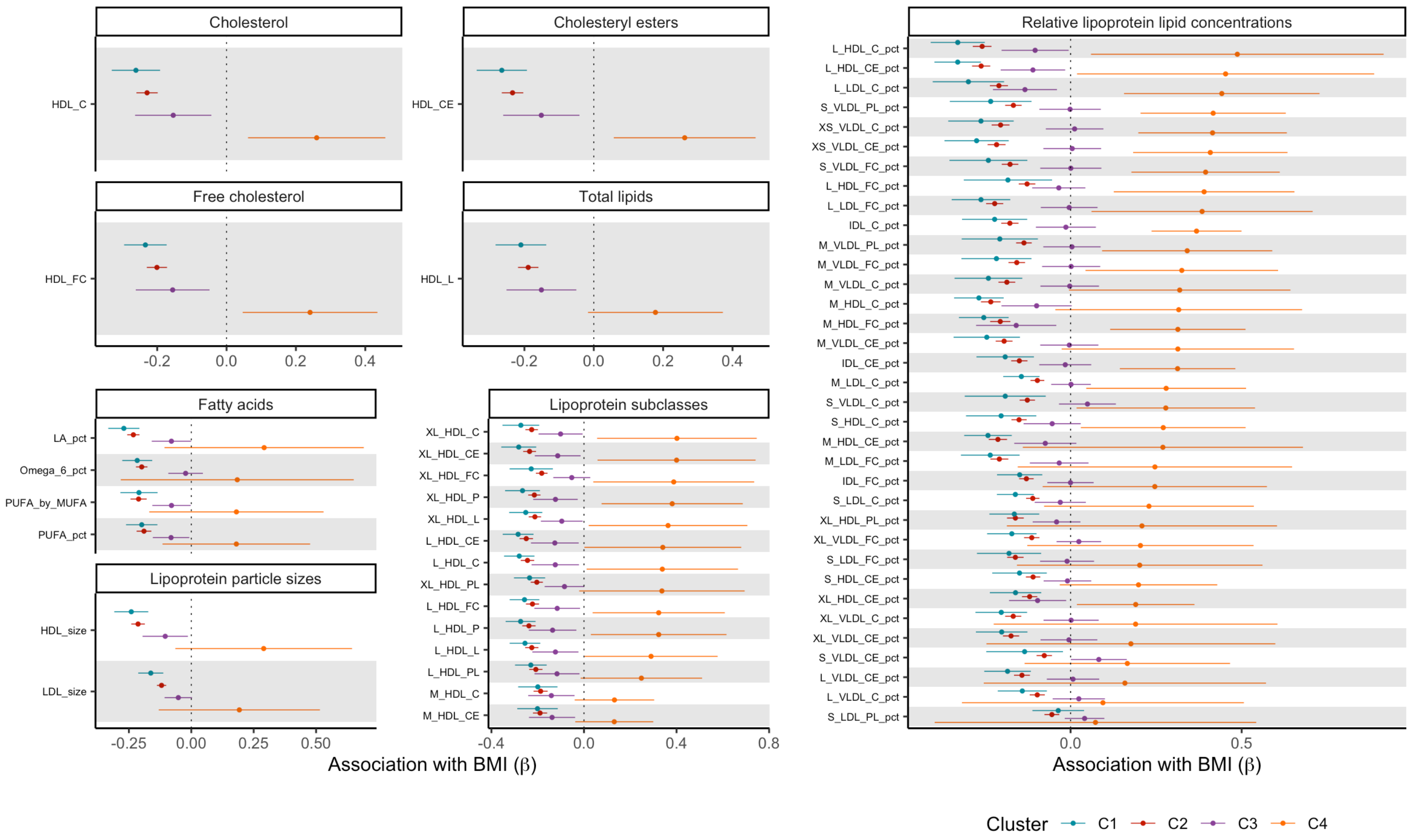

Supplementary Figure 4. NMR metabolites with decreased T2D risk and their cluster-specific association with BMI disaggregated by metabolite group. *IVW MR estimates for the effect of different BMI clusters on metabolite levels for a subset of metabolites with the most significant between-cluster heterogeneity. Points and horizontal bars represent the IVW MR estimates and 95% confidence intervals, respectively. These estimates were obtained from combined, meta-analysed data from the UK Biobank and Estonian Biobank (N = 619,372). Colours indicate the four clusters: cyan = C1 (high-risk), red = C2 (medium-risk), lilac = C3 (null), and orange = C4 (protective). For full names of metabolites see Supplementary Data 9.*

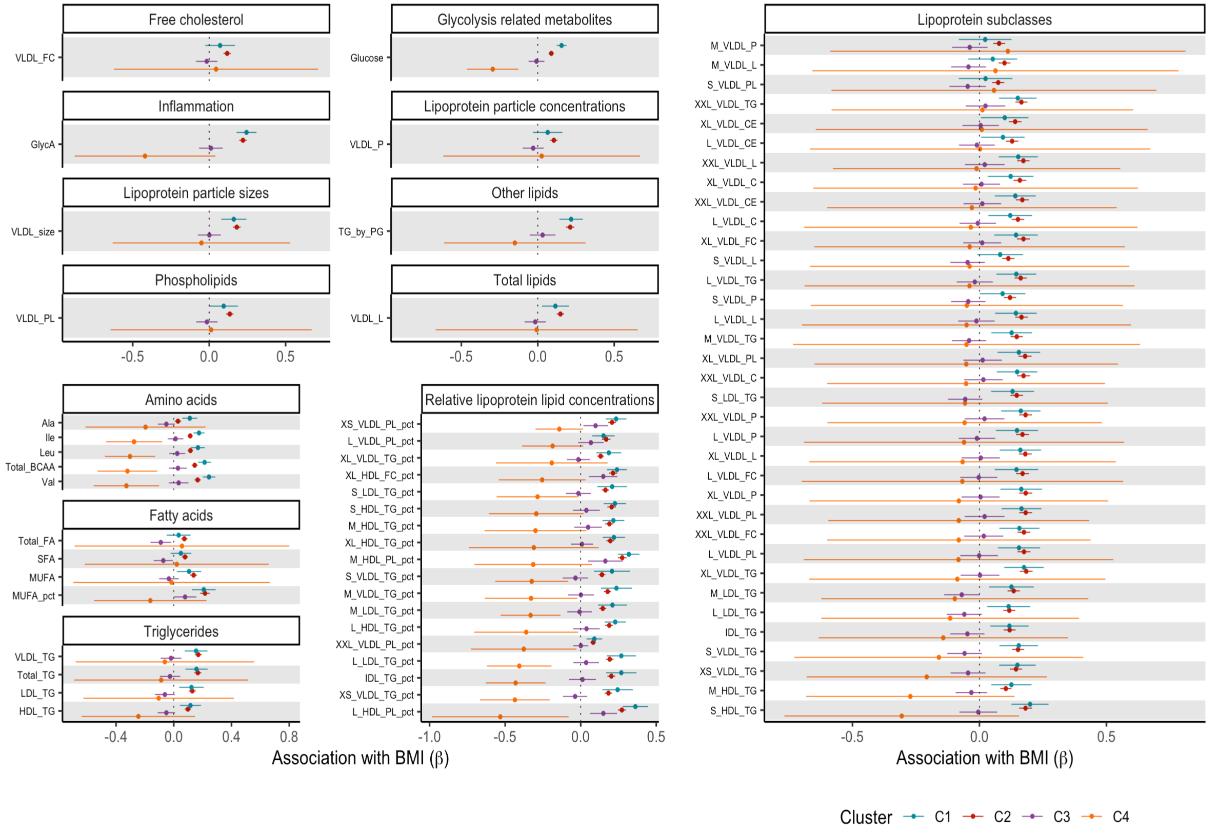

Supplementary Figure 5. NMR metabolites with increased T2D risk and their cluster-specific association with BMI disaggregated by metabolite group.

IVW MR estimates for the effect of different BMI clusters on metabolite levels for a subset of metabolites with the most significant between-cluster heterogeneity. Points and horizontal bars represent the IVW MR estimates and 95% confidence intervals, respectively. These estimates were obtained from combined, meta-analysed data from the UK Biobank and Estonian Biobank (N = 619,372). Colours indicate the four clusters: cyan = C1 (high-risk), red = C2 (medium-risk), lilac = C3 (null), and orange = C4 (protective). For full names of metabolites see Supplementary Data 9. For full names of metabolites see Supplementary Data 9.

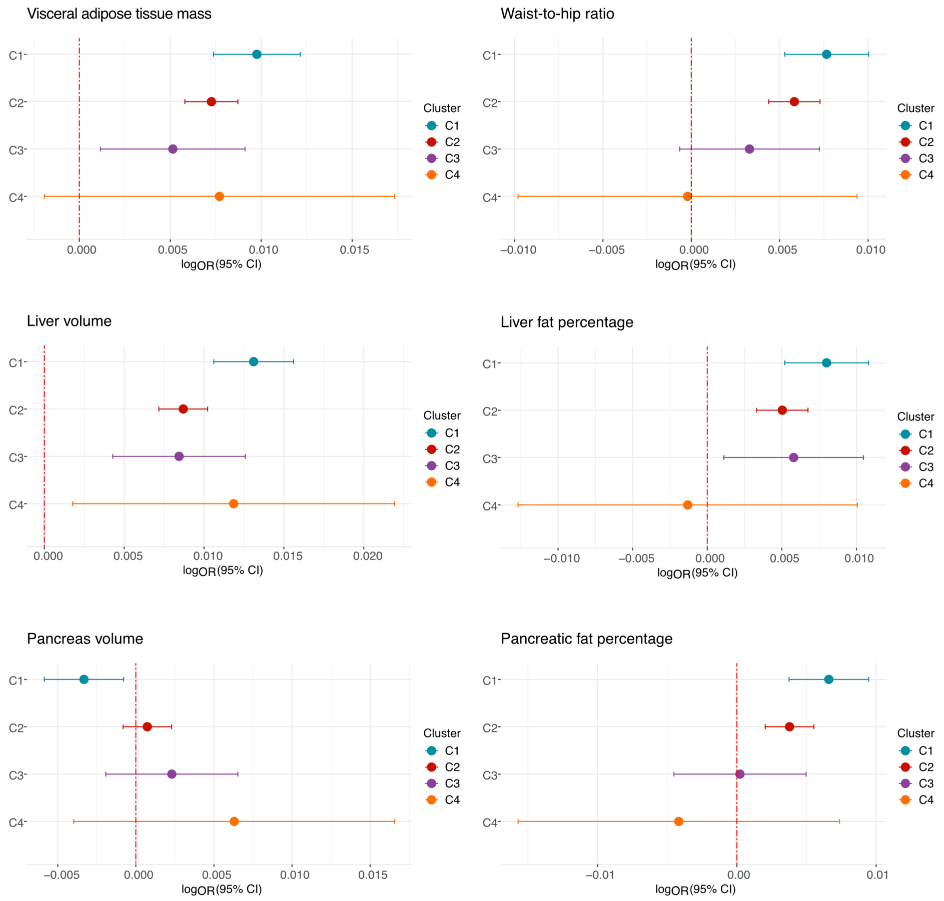

Supplementary Figure 6. Cluster-specific associations between BMI and imaging-derived traits.

Forest plots showing IVW Mendelian randomization (MR) estimates expressed as odds ratios (ORs) with 95% confidence intervals (CIs) for the associations between BMI and imaging-derived traits in UK Biobank participants. The traits include waist-to-hip ratio (N = 35,780), visceral adipose tissue mass (N = 35,591), liver fat percentage (N = 27,732), liver volume (N = 35,591), pancreatic fat percentage (N = 26,942), and pancreatic volume (N = 34,393). The figure presents MR estimates obtained using cluster-specific MR instruments. Colors indicate the four clusters: cyan = C1 (high-risk), red = C2 (medium-risk), lilac = C3 (null), and orange = C4 (protective).
