# Supplementary material for: Genetic and molecular signatures highlight diverse pathways linking obesity to type 2 diabetes
Source: Nat Commun. 2026 Jul 8;17:5680. doi: 10.1038/s41467-026-74675-9 (PMC13347065; doi:10.1038/s41467-026-74675-9)
Supplement: Supplementary file 2 — Description of Additional Supplementary Files [file 41467_2026_74675_MOESM2_ESM.pdf]

Description of Additional Supplementary Files

Supplementary Data 1
Description: GWAS summary statistics for SNPs that are significant in the GIANT + FinnGen meta-analysis for BMI, their effect on T2D, and the clustering results.

Supplementary Data 2
Description: Enriched functional terms among genes annotated (using the SNP2GENE function in FUMA) to SNPs belonging to Cluster 1 (High-risk).

Supplementary Data 3
Description: Enriched functional terms among genes annotated (using the SNP2GENE function in FUMA) to SNPs belonging to Cluster 2 (Medium-risk).

Supplementary Data 4
Description: Enriched functional terms among genes annotated (using the SNP2GENE function in FUMA) to SNPs belonging to Cluster 3 (Null).

Supplementary Data 5
Description: Enriched functional terms among genes annotated (using the SNP2GENE function in FUMA) to SNPs belonging to Cluster 4 (Protective).

Supplementary Data 6
Description: Mendelian randomization (MR) results for the effect of each BMI cluster on proteins.

Supplementary Data 7
Description: MR results for the effect of each BMI cluster on proteins and for the effect of proteins on T2D.

Supplementary Data 8
Description: Mendelian randomization (MR) results for the effect of each BMI cluster on NMR metabolites.

Supplementary Data 9
Description: MR results for the effect of each BMI cluster on NMR metabolites and for the effect of NMR metabolites on T2D.

Supplementary Data 10
Description: MR results for the effects of each BMI cluster on NMR metabolites, restricted to significant findings only.

Supplementary Data 11
Description: Mediation results for NMR metabolites using the product-of-coefficients method for each biomarker by BMI cluster.

Supplementary Data 12
Description: MR results for the effect of each BMI cluster on clinical biomarkers, including glycaemic traits.

Supplementary Data 13
Description: MR results for the effect of each BMI cluster on clinical biomarkers, including glycaemic traits, and for the effect of clinical biomarkers on T2D.

Supplementary Data 14
Description: Mediation results for clinical biomarkers using the product-of-coefficients method for each biomarker by BMI cluster.
