# Supplementary material for: Genetic and molecular signatures highlight diverse pathways linking obesity to type 2 diabetes
Source: Nat Commun. 2026 Jul 8;17:5680. doi: 10.1038/s41467-026-74675-9 (PMC13347065; doi:10.1038/s41467-026-74675-9)
Supplement: Supplementary file 4 — Reporting Summary [file 41467_2026_74675_MOESM4_ESM.pdf]

nature portfolio

Corresponding author(s): Åsa Johansson

Last updated by author(s): 4/18/2026

Reporting Summary

Nature Portfolio wishes to improve the reproducibility of the work that we publish. This form provides structure for consistency and transparency in reporting. For further information on Nature Portfolio policies, see our Editorial Policies and the Editorial Policy Checklist.

Statistics

For all statistical analyses, confirm that the following items are present in the figure legend, table legend, main text, or Methods section.

n/a	Confirmed	
☐	☒	The exact sample size (n) for each experimental group/condition, given as a discrete number and unit of measurement
☐	☒	A statement on whether measurements were taken from distinct samples or whether the same sample was measured repeatedly
☐	☒	The statistical test(s) used AND whether they are one- or two-sided<br>*Only common tests should be described solely by name; describe more complex techniques in the Methods section.*
☐	☒	A description of all covariates tested
☐	☒	A description of any assumptions or corrections, such as tests of normality and adjustment for multiple comparisons
☐	☒	A full description of the statistical parameters including central tendency (e.g. means) or other basic estimates (e.g. regression coefficient) AND variation (e.g. standard deviation) or associated estimates of uncertainty (e.g. confidence intervals)
☐	☒	For null hypothesis testing, the test statistic (e.g. F, t, r) with confidence intervals, effect sizes, degrees of freedom and P value noted<br>*Give P values as exact values whenever suitable.*
☒	☐	For Bayesian analysis, information on the choice of priors and Markov chain Monte Carlo settings
☒	☐	For hierarchical and complex designs, identification of the appropriate level for tests and full reporting of outcomes
☒	☐	Estimates of effect sizes (e.g. Cohen's d, Pearson's r), indicating how they were calculated

Our web collection on statistics for biologists contains articles on many of the points above.

Software and code

Policy information about availability of computer code

Data collection	All data used in this study were collected by the UK Biobank and provided to scientists upon application using the relevant provided channels. Other data are from public repositories. Details have been provided in the manuscript
Data analysis	All analyses were conducted using open source statistical software, R version 4.2 or PLINK v1.9. All custom code is available in GitHub/Zenodo

For manuscripts utilizing custom algorithms or software that are central to the research but not yet described in published literature, software must be made available to editors and reviewers. We strongly encourage code deposition in a community repository (e.g. GitHub). See the Nature Portfolio guidelines for submitting code & software for further information.

Data

Policy information about availability of data

All manuscripts must include a data availability statement. This statement should provide the following information, where applicable:

- Accession codes, unique identifiers, or web links for publicly available datasets
- A description of any restrictions on data availability
- For clinical datasets or third party data, please ensure that the statement adheres to our policy

The UKB data used in this study are subject to controlled access due to the inclusion of sensitive participant information. Data are available to bona fide researchers upon application to the UKB via the Access Management System [https://www.ukbiobank.ac.uk]. Access is granted following review and approval of a research proposal and is subject to a data use agreement that restricts data use to the approved project and prohibits re-identification of participants. The UKB typically

responds to access requests within a defined review period as outlined on their website. BMI summary statistics data are from FinnGen [https://www.finngen.fi/en/access_results], file: summary_stats_finngen_R9_BMI_IRN.gz and GIANT [https://portals.broadinstitute.org/collaboration/giant/index.php/GIANT_consortium_data_files#GWAS_Anthropometric_2015_BMI_Summary_Statistics], file: SNP_gwas_mc_merge_nogc.tbl.uniq.gz. T2D data were downloaded from DIAGRAM [https://diagram-consortium.org] using the file: Mahajan.NatGenet2018b.T2D-noUKBB.European.txt. The pQTL data were obtained from the discovery credible sets in the UKB as reported in Sun B. et al. (ST16)31. Data for NMR metabolites were downloaded from GWAS catalogue accession numbers GCST90449363 - GCST90451603 (full summary statistics) and Zenodo [https://dx.doi.org/10.5281/zenodo.13937265)] (significant lead SNPs). Data for clinical biomarkers were obtained from Sinnott-Armstrong et al.33 [https://doi.org/10.35092/yhjc.12355382] for the 35 biomarkers available in the UKB, and from the MAGIC consortium [https://magicinvestigators.org] for glycaemic traits. GWAS summary statistics for imaging-derived traits and WHR are available at Zenodo [https://doi.org/10.5281/zenodo.19590199].

Research involving human participants, their data, or biological material

Policy information about studies with human participants or human data. See also policy information about sex, gender (identity/presentation), and sexual orientation and race, ethnicity and racism.

Reporting on sex and gender

We used sex (biological attribute) as a major covariate in this study which was adjusted for in all individual level analyses. We also conducted sex-stratified analyses which we report the outcomes in the manuscript. Sex was self-reported in the UK Biobank or determined genetically. In cases where there was discrepancy between self-reported and genetically determined sex, or aneuploidy, participants were excluded from the analyses.

Reporting on race, ethnicity, or other socially relevant groupings

We used data from participants of European ancestry in our analyses owing to the fact that we used genetic data which is prone to confounding by population structure. We detail this in the manuscript.

Population characteristics

The demographic characteristics used in this study included age at enrollment(in years), sex, and further lifestyle characteristics like smoking, alcohol consumption and anthropometric measurements (body mass index, BMI). We also used individual genetic data and related covariates: principal components of population structure and the genetic batch an individual's sample was genotyped in. We have described this extensively in the manuscript.

Recruitment

Enrollment in the UK Biobank was done via invitation by mail to 22 recruitment centres where interviews were conducted, participants filled questionnaires and anthropometric measurements and biological samples were collected. We are aware of and acknowledge reports on healthy volunteer and self-selection biases as well as demographic skew(non-representative of general population) in the UK Biobank. We comment on generalizability of our results and the applicability of genetic clusters.

Ethics oversight

The data used in this project are under UKB application number 15152. Further ethics approval was obtained from the Swedish EPM, number 2020-04415.

Note that full information on the approval of the study protocol must also be provided in the manuscript.

Field-specific reporting

Please select the one below that is the best fit for your research. If you are not sure, read the appropriate sections before making your selection.

☒ Life sciences ☐ Behavioural & social sciences ☐ Ecological, evolutionary & environmental sciences

For a reference copy of the document with all sections, see nature.com/documents/nr-reporting-summary-flat.pdf

Life sciences study design

All studies must disclose on these points even when the disclosure is negative.

Sample size

We used the entire UK Biobank resource sample and systematically chose participants with attributes of interest to arrive at a final sample size. In analyses using summary GWAS data, we combined two cohorts to increase sample size and power. In both cases, the resultant sample sizes were deemed sufficient being higher than recommendations based on covariate numbers.

Data exclusions

We excluded participants were related to avoid bias in analyses using genetic data. We also excluded participants who were missing the exposure of interest, BMI. We have provided details on exclusion in the manuscript.

Replication

Experiments were not replicated or performed independently.

Randomization

Randomization was not relevant to this study as it was not designed to be a randomized trial.

Blinding

Investigators were not blinded

Reporting for specific materials, systems and methods

We require information from authors about some types of materials, experimental systems and methods used in many studies. Here, indicate whether each material, system or method listed is relevant to your study. If you are not sure if a list item applies to your research, read the appropriate section before selecting a response.

Materials & experimental systems

n/a	Involved in the study
☒	☐ Antibodies
☒	☐ Eukaryotic cell lines
☒	☐ Palaeontology and archaeology
☒	☐ Animals and other organisms
☒	☐ Clinical data
☒	☐ Dual use research of concern
☒	☐ Plants

Methods

n/a	Involved in the study
☒	☐ ChIP-seq
☒	☐ Flow cytometry
☒	☐ MRI-based neuroimaging

Plants

Seed stocks

Report on the source of all seed stocks or other plant material used. If applicable, state the seed stock centre and catalogue number. If plant specimens were collected from the field, describe the collection location, date and sampling procedures.

Novel plant genotypes

Describe the methods by which all novel plant genotypes were produced. This includes those generated by transgenic approaches, gene editing, chemical/radiation-based mutagenesis and hybridization. For transgenic lines, describe the transformation method, the number of independent lines analyzed and the generation upon which experiments were performed. For gene-edited lines, describe the editor used, the endogenous sequence targeted for editing, the targeting guide RNA sequence (if applicable) and how the editor was applied.

Authentication

Describe any authentication procedures for each seed stock used or novel genotype generated. Describe any experiments used to assess the effect of a mutation and, where applicable, how potential secondary effects (e.g. second site T-DNA insertions, mosiacism, off-target gene editing) were examined.
