# Supplementary material for: Genetic and molecular signatures highlight diverse pathways linking obesity to type 2 diabetes
Source: Nat Commun. 2026 Jul 8;17:5680. doi: 10.1038/s41467-026-74675-9 (PMC13347065; doi:10.1038/s41467-026-74675-9)
Supplement: Supplementary file 5 — Transparent Peer Review file [file 41467_2026_74675_MOESM5_ESM.pdf]

nature portfolio

Peer Review File

Genetic and molecular signatures highlight diverse pathways linking obesity to type 2 diabetes

Corresponding Author: Professor Åsa Johansson

This file contains all reviewer reports in order by version, followed by all author rebuttals in order by version.

Version 0:

Reviewer comments:

Reviewer #1

(Remarks to the Author)
The authors investigated the association between BMI and T2D using cluster Mendelian Randomization, and combined this with additional proteomics analyses to identify potential mediating proteins of the identified clusters with T2D. This study is of interest given the consistent interest by the research community in the heterogeneity in the association between BMI and T2D. The authors generally wrote a clear manuscript and used common methodology and reporting. Nevertheless, I have a number of comments for further improvement of the manuscript.

- While the cluster Mendelian Randomization analysis is of interest, and the authors performed replication in an independent cohort (and performed analyses sex-specific), the readers still get little insight into the biomolecular mechanisms involved. Despite the authors added the proteomics analyses, the identified proteins between cluster 1 and 2 are largely overlapping. I would therefore like to propose the authors performed additional bioinformatics analyses. The SNPs in the different clusters can be mapped to genes, which can be used in pathway analyses, etc. This would give us the opportunity to get more insights into the potential distinct biological mechanisms driving these clusters. Also based on the proteins identified to be associated with the different clusters it would be helpful to perform additional bioinformatics analyses to identify the relevant pathways. This would make the interpretation of the findings easier (also given a number of the proteins identified by the authors are not generally known).
- While the authors interpret the results as potential mediation, the authors did not perform a formal mediation analysis. It is therefore strongly recommended to perform a formal mediation analysis, using the two-step approach for mediation in Mendelian Randomization (see for example: https://link.springer.com/article/10.1007/s10654-021-00757-1). This would also provide the reader with more insights into the mediating effects of the identified proteins.
- The associations between the BMI-cluster PRSs and the protein levels are not performed using conventional two-sample MR methodology, and I wonder whether any of these associations could be potentially harmed by directional pleiotropy.
- It would be helpful to test whether the clusters on T2D are harmed by directional pleiotropy.
- The authors did use the full UK biobank sample, but there are also some familial relationships in UK Biobank. I would like to recommend the authors to perform sensitivity analyses excluding related individuals.
- From the 513 independent SNPs identified in the GIANT-Finngen meta-analysis, only 463 SNPs were available in DIAGRAM. Did the authors consider the identification of proxy SNPs?
- In addition to T2D, it would also be helpful to use the glycemic measures as additional outcomes. measures like insulin and homa-ir (amongst the others) might be helpful to further interpret the results.

minor comments:
- Having the x-axis of the figures presented in supplementary figure 2 on a logscale is more appropriate I think

Reviewer #2

(Remarks to the Author)
The manuscript offers a novel and insightful exploration of the genetic and proteomic pathways linking obesity to Type 2 diabetes (T2D), employing MR-Clust to cluster genetic variants based on their impacts on BMI and T2D. The authors identify four clusters of SNPs and constructed cluster-specific polygenic scores to identify significant associations with hundreds proteins measured in the UKB. Of these, eight proteins were shown to be causally associated with T2D using MR. This methodological approach is innovative and enhances our understanding of the genetic underpinnings of these conditions. At

the same time, the broader mechanisms captured by the clusters themselves were not fully clear, and enthusiasm was dampened by the narrow focus on genetic and proteomic data without substantial integration of clinical outcomes. These gaps limited the impact and potential translation these findings into practical applications for disease management and treatment.

Strengths noted:
• Well-written with clear descriptions of methods
• Cutting-edge statistical genetic analyses
• Linking genetic findings to proteomic data

Limitations:
• Focus on genetic and proteomic data without substantial integration of clinical outcomes
• Inclusion of a single dataset (UKB) without additional replication
• The value gained from identifying the clusters themselves was not completely clear, for example in terms of the biological relevance of each cluster. This is particularly important for justifying the initial clustering approach.

To elevate the quality and impact of the manuscript, here are some suggestions:

1. Comprehensive clinical characterization: It would be highly beneficial to include detailed clinical data analyses similar to those found in the study by Coral et al. (Nat Metab. 2023 Feb;5(2):237-247. doi: 10.1038/s42255-022-00731-5), which successfully integrated genetic data with extensive metabolic and clinical phenotyping. This approach could involve correlating the identified genetic clusters with a range of clinical outcomes, such as insulin resistance, glucose tolerance tests, body composition (including fat distribution) and detailed lipid profiles, to provide a clearer picture of how these genetic predispositions manifest in observable clinical phenotypes.

2. Characterization of individuals based on extremes of BMI-PRSs: Further analysis could focus on individuals at the extremes of the polygenic risk score spectrum. This approach would help identify the most and least vulnerable subpopulations to T2D among those classified as obese, providing deeper insights into the preventive and therapeutic potentials targeted to specific genetic profiles.

3. Inclusion of longitudinal clinical data: Including longitudinal data to observe disease progression (including metabolic complications of obesity) in individuals across different genetic risk profiles would be invaluable. This data could help in understanding the temporal dynamics of T2D development and the potential long-term effects of these genetic factors on health outcomes.

4. Additional investigation into the biological relevance of each cluster. For example, pathway analysis of proteins associated with each cluster may provide a broader understanding of potential underlying mechanisms.

5. More direct comparison of its findings to those of relevant studies, such as the one by Coral et al. mentioned above. Such comparisons would not only contextualize findings within the existing literature, but also highlight the novel contributions of the study in comparison to established research.

In conclusion, while the research presented is promising and methodologically sound, the addition of analyses to demonstrate broader biological and clinical relevance of the clusters would substantially increase its relevance and impact.

Reviewer #3

(Remarks to the Author)
I co-reviewed this manuscript with one of the reviewers who provided the listed reports. This is part of the Nature Communications initiative to facilitate training in peer review and to provide appropriate recognition for Early Career Researchers who co-review manuscripts.

Version 1:

Reviewer comments:

Reviewer #1

(Remarks to the Author)
The authors addressed my comments and concerns, and the paper is now suitable for publication.

(Remarks on code availability)
Website seems empty

Reviewer #2

(Remarks to the Author)
The authors have clearly invested a great deal of effort in revising the manuscript, and it is considerably strengthened. In particular, expanding beyond proteomics to include additional phenotypes (NMR metabolomics and multiple clinical

biomarkers, including glycemic traits and lipid-related measures) substantially improved the characterization of the clusters. It was also appreciated that the authors added to the discussion a comparison of the present study to prior work investigating different obesity profiles in relation to T2D risk.

While the manuscript involves well-performed analyses and methodological rigor, it would be ideal for the following two goals to be accomplished 1) mechanistic/molecular interpretability of the clusters (i.e. what pathways(s) are being captured?) and 2) clinical/pathophysiological interpretability (i.e. are these distinct clinical subtypes?). Both are of these are addressed to some extent, and the new analyses are very helpful toward these goals, but I still came away from reading the manuscript unsure as to what the clusters truly represented mechanistically and clinically. For example, how is Cluster 1 meaningfully different from Cluster 2?

It is possible that consideration of other key physiological or anatomical dimensions of obesity heterogeneity (e.g., body fat distribution and ectopic fat measures, imaging-derived traits, or more direct indices of insulin resistance and beta-cell function) could help. A more systematic phenome-wide characterization might help clarify whether the identified clusters correspond to distinct clinical patterns and ideally might help further differentiate Cluster 1 and 2.

Regarding longitudinal data, the authors' point that longitudinal molecular datasets with sufficient sample size are currently limited is well-taken. However, it may be possible to look at longitudinal clinical follow-up (even without repeated molecular profiling), for example how cluster-specific PRS are associated with trajectories of glycemic measures, weight, or incident cardiometabolic outcomes. Such analyses might further strengthen the translational relevance and help support the broader claims about divergent pathways from obesity to T2D. These analyses are not critical, but are just suggestions if they might help paint a clearer picture as to what these clusters represent clinically.

Overall, the revision substantially strengthens the work and resolves several of the concerns raised previously. The remaining (unfortunately large) limitation is that the clusters are still challenging to fully interpret in terms of both the pathways/disease mechanisms they capture and any clinical application/relevance (particularly with regard to understanding how Cluster 1 differs from Cluster 2).

(Remarks on code availability)

Open Access This Peer Review File is licensed under a Creative Commons Attribution 4.0 International License, which permits use, sharing, adaptation, distribution and reproduction in any medium or format, as long as you give appropriate credit to the original author(s) and the source, provide a link to the Creative Commons license, and indicate if changes were made.
In cases where reviewers are anonymous, credit should be given to 'Anonymous Referee' and the source.
The images or other third party material in this Peer Review File are included in the article's Creative Commons license, unless indicated otherwise in a credit line to the material. If material is not included in the article's Creative Commons license and your intended use is not permitted by statutory regulation or exceeds the permitted use, you will need to obtain permission directly from the copyright holder.
To view a copy of this license, visit https://creativecommons.org/licenses/by/4.0/

We thank the reviewers for their valuable and constructive feedback and have revised the manuscript accordingly. Below, we provide a detailed point-by-point response to the reviewers' comments. The additional and revised analyses suggested by the reviewers have added substantial value to the manuscript. Most notably, we have expanded the scope of the study by including additional molecular traits beyond proteomics; the revised manuscript now also incorporates metabolomics data as well as clinical biomarkers, including glycaemic traits. These newly included traits show stronger and clearer results compared with analyses based solely on proteins. Consequently, the manuscript has undergone substantial revision and, in our view, has been significantly improved as a result.

REVIEWER COMMENTS

Reviewer #1 (Remarks to the Author):

The authors investigated the association between BMI and T2D using cluster Mendelian Randomization, and combined this with additional proteomics analyses to identify potential mediating proteins of the identified clusters with T2D. This study is of interest given the consistent interest by the research community in the heterogeneity in the association between BMI and T2D. The authors generally wrote a clear manuscript and used common methodology and reporting. Nevertheless, I have a number of comments for further improvement of the manuscript.

- While the cluster Mendelian Randomization analysis is of interest, and the authors performed replication in an independent cohort (and performed analyses sex-specific), the readers still get little insight into the biomolecular mechanisms involved. Despite the authors added the proteomics analyses, the identified proteins between cluster 1 and 2 are largely overlapping. I would therefore like to propose the authors performed additional bioinformatics analyses. The SNPs in the different clusters can be mapped to genes, which can be used in pathway analyses, etc. This would give us the opportunity to get more insights into the potential distinct biological mechanisms driving these clusters. Also based on the proteins identified to be associated with the different clusters it would be helpful to perform additional bioinformatics analyses to identify the relevant pathways. This would make the interpretation of the findings easier (also given a number of the proteins identified by the authors are not generally known).

*Response **R1:1**: We thank the reviewer for their comments. To address this, we used FUMA to map all genes associated with all the SNPs in each cluster and included analyses for differentially expressed genes in different tissues. We further used g:Profiler to perform detailed pathway, molecular and biological function analyses for each respective cluster This is now described in the "Gene mapping and enrichment analysis of cluster-specific SNPs" paragraph in the Results section and Supplementary Data 2-5. In addition, we performed enrichment analyses based on proteins that were differentially affected by the clusters; these analyses are now described in the text and illustrated in Figure 4.*

- While the authors interpret the results as potential mediation, the authors did not perform a formal mediation analysis. It is therefore strongly recommended to perform a formal mediation analysis, using the two-step approach for mediation in Mendelian Randomization (see for example: https://link.springer.com/article/10.1007/s10654-021-00757-1). This would also provide the reader with more insights into the mediating effects of the identified proteins.

Response R1:2*: We thank the reviewer for this comment and have now conducted formal mediation analyses using the two-step approach suggested, for each cluster. We provide the details of the approach in Methods section and report the results in the Results section in association with describing the MR results for the different molecular traits. Results are also provided in Supplementary Data 10 and 13.*

- The associations between the BMI-cluster PRSs and the protein levels are not performed using conventional two-sample MR methodology, and I wonder whether any of these associations could be potentially harmed by directional pleiotropy.
- It would be helpful to test whether the clusters on T2D are harmed by directional pleiotropy.

Response R1:3 *We have now revised the analyses and instead performed a traditional two-sample MR of the association between BMI clusters and protein levels using individual SNPs rather than combined into a PRS (as in the previous version). We then also performed MR-egger analyses to assess for horizontal pleiotropy and report these results and additionally provide comprehensive details in the supplementary materials for the respective molecular traits (Supplementary Data 7,8, and 11).*

- The authors did use the full UK biobank sample, but there are also some familial relationships in UK Biobank. I would like to recommend the authors to perform sensitivity analyses excluding related individuals.

Response R1:4: *Most analyses have been conducted using unrelated individuals and restricted to participants self-reporting as European ancestry. We apologize if this was not clear in the previous version and have now revised it to clarify this point. The only exception was the NMR metabolite analyses where we used summary statistics from previous GWAS studies and for these analyses, no such results were available.*

- From the 513 independent SNPs identified in the GIANT-Finngen meta-analysis, only 463 SNPs were available in DIAGRAM. Did the authors consider the identification of proxy SNPs?

Response R1:5 *We apologise for the lack of clarity and have now revised the text. All 513 SNPs were indeed available in DIAGRAM. However, after harmonization of the exposure and outcome datasets, only 463 passed meaning they were suitable for subsequent analyses. There is a built-in search for proxies in this process, so proxies had been considered. For SNPs that were excluded at this stage, we have now also searched for all possible proxies with $r2 \geq 0.8$ and iteratively replaced them in the exposure file and harmonized again. This process yielded three additional SNPs. Given the small number, we did not revise the analyses to include them and hope this is acceptable to the reviewer.*

- In addition to T2D, it would also be helpful to use the glycemic measures as additional outcomes. measures like insulin and homa-ir (amongst the others) might be helpful to further interpret the results.

Response R1:5 *We thank the reviewer for this valuable suggestion which led us to include glycaemic traits but also metabolomics and other blood/urine clinical biomarkers alongside proteomics, resulting in stronger, clearer results and substantial improvement of the manuscript. These additions have led to substantive changes throughout the manuscript and the supplementary material.*

minor comments:
- Having the x-axis of the figures presented in supplementary figure 2 on a logscale is more appropriate I think

> ***Response R1:6*** *We thank the reviewer and have changed accordingly. Figures now show either the MR IVW estimate or the log OR/HR.*

Reviewer #2 (Remarks to the Author):

The manuscript offers a novel and insightful exploration of the genetic and proteomic pathways linking obesity to Type 2 diabetes (T2D), employing MR-Clust to cluster genetic variants based on their impacts on BMI and T2D. The authors identify four clusters of SNPs and constructed cluster-specific polygenic scores to identify significant associations with hundreds proteins measured in the UKB. Of these, eight proteins were shown to be causally associated with T2D using MR. This methodological approach is innovative and enhances our understanding of the genetic underpinnings of these conditions. At the same time, the broader mechanisms captured by the clusters themselves were not fully clear, and enthusiasm was dampened by the narrow focus on genetic and proteomic data without substantial integration of clinical outcomes. These gaps limited the impact and potential translation these findings into practical applications for disease management and treatment.

Strengths noted:
• Well-written with clear descriptions of methods
• Cutting-edge statistical genetic analyses
• Linking genetic findings to proteomic data

Limitations:
• Focus on genetic and proteomic data without substantial integration of clinical outcomes
• Inclusion of a single dataset (UKB) without additional replication
• The value gained from identifying the clusters themselves was not completely clear, for example in terms of the biological relevance of each cluster. This is particularly important for justifying the initial clustering approach.

To elevate the quality and impact of the manuscript, here are some suggestions:

1. Comprehensive clinical characterization: It would be highly beneficial to include detailed clinical data analyses similar to those found in the study by Coral et al. (Nat Metab. 2023 Feb;5(2):237-247. doi: 10.1038/s42255-022-00731-5), which successfully integrated genetic data with extensive metabolic and clinical phenotyping. This approach could involve correlating the identified genetic clusters with a range of clinical outcomes, such as insulin resistance, glucose tolerance tests, body composition (including fat distribution) and detailed lipid profiles, to provide a clearer picture of how these genetic predispositions manifest in observable clinical phenotypes.

> ***Response R2:1*** *We thank the reviewer for this suggestion which has dramatically improved the manuscript. We agree with the reviewer that including only proteomics data might be less informative than incorporating a broader range of potential*

intermediate phenotypes. A recent study focusing mainly on body composition was recently published (PMID: 38530928) and since body composition is also more of a general indicator than a biologically plausible mediator, we have focused on including molecular data only. This includes the plasma proteome (as in the previous version), as well as the metabolome (250 NMR measures) and clinical biomarkers, including glycaemic traits and other plasma and urine biomarkers also including lipid data. These additions have led to substantive changes throughout the manuscript and the supplementary material.

2. Characterization of individuals based on extremes of BMI-PRSs: Further analysis could focus on individuals at the extremes of the polygenic risk score spectrum. This approach would help identify the most and least vulnerable subpopulations to T2D among those classified as obese, providing deeper insights into the preventive and therapeutic potentials targeted to specific genetic profiles.

*Response R2:2: We agree with the reviewer, and this was indeed one of the initial aims of the current project and the reason for focusing on PRS rather than designing a standard MR-study. However, despite the large number of SNPs identified in recent GWAS, when focusing on cohorts that do not overlap with UK Biobank (to allow evaluation in an independent cohort) and dividing the variants into clusters, there were relatively few variants per cluster (see Table 1). This means that, while the effect of each cluster (e.g., the increased or decreased risk of T2D per 1 SD increase in a cluster-specific BMI PRS; Table 1 and Figure 3a) is substantial, there are too few SNPs in most clusters to identify individuals with a 1 SD unit increase in BMI due to the PRS for any single cluster (*Supplementary Figure 2*). Therefore, the analysis suggested by the reviewer is unfortunately not as informative as we had hoped. We have, however, now included cumulative incidence rates for T2D among individuals in the top half and bottom half of the BMI PRS for the different clusters to further illustrate potential differences (Figure 3b). It should, however, be noted, as outlined in the text, that the top and bottom half for the protective cluster is associated with a much smaller difference in BMI (due to the limited number of SNPs, Supplementary Figure 2) compared to the harmful clusters, particularly the less harmful cluster, which contains more SNPs and thus allows better differentiation between individuals. With the continued growth of available GWAS datasets, we foresee that the suggested analysis will be feasible within a few years or less.*

3. Inclusion of longitudinal clinical data: Including longitudinal data to observe disease progression (including metabolic complications of obesity) in individuals across different genetic risk profiles would be invaluable. This data could help in understanding the temporal dynamics of T2D development and the potential long-term effects of these genetic factors on health outcomes.

Response R2:3 *We thank the reviewer for this insightful suggestion. We agree that inclusion of longitudinal molecular data would be extremely valuable for understanding disease progression and the long-term effects of genetic risk factors. However, currently there is very limited longitudinal molecular data available, and given that our study's power is already constrained (e.g., for proteomics with ~50,000 individuals), it is unfortunately not feasible to perform such analyses at this time. We*

hope that as larger longitudinal molecular datasets become available in the coming years, these important analyses will be possible.

4. Additional investigation into the biological relevance of each cluster. For example, pathway analysis of proteins associated with each cluster may provide a broader understanding of potential underlying mechanisms.

> ***Response R2:4***: *While we did not observe a very strong differentiation in the proteins associated with the different clusters, particularly not when requiring that proteins affected by each respective cluster should also be associated with T2D, we agree with this Reviewer (and Reviewer #1) that the biological relevance of the clusters was not very evident in the previous version of the manuscript. In line with our response to Reviewer #1, we have now performed enrichment analyses for the different clusters (see Response R1:1). These results are included in the revised manuscript and provide additional biological context and relevance for the clusters. Moreover, as suggested above, we have also incorporated additional biomarkers beyond proteomics. Interestingly, these new data show a much clearer differentiation between the clusters, and the revised version therefore offers a more comprehensive understanding of the underlying mechanisms.*

5. More direct comparison of its findings to those of relevant studies, such as the one by Coral et al. mentioned above. Such comparisons would not only contextualize findings within the existing literature, but also highlight the novel contributions of the study in comparison to established research.

> ***Response R2:5*** *We have now rewritten the discussion section and compared our findings with previous studies that specifically investigated the causal links between different obesity profiles and T2D. We compare the work of, for example, Coral et al and Abrahams.*

In conclusion, while the research presented is promising and methodologically sound, the addition of analyses to demonstrate broader biological and clinical relevance of the clusters would substantially increase its relevance and impact.

> ***Response R2:5*** *We fully agree that demonstrating broader biological and clinical relevance is important, and we have therefore implemented the suggested additional analyses in the revised manuscript. We hope that the reviewer finds these revisions satisfactory. We also wish to express our sincere appreciation to both this reviewer and Reviewer #1, the insightful suggestions from both have substantially strengthened and enhanced the overall impact of our study.*

Reviewer #3 (Remarks to the Author):

I co-reviewed this manuscript with one of the reviewers who provided the listed reports. This is part of the Nature Communications initiative to facilitate training in peer review and to provide appropriate recognition for Early Career Researchers who co-review manuscripts.

Point-to-Point response to reviewers' comments

REVIEWERS' COMMENTS

Reviewer #1 (Remarks to the Author):

The authors addressed my comments and concerns, and the paper is now suitable for publication.

Reviewer #1 (Remarks on code availability):

Website seems empty

Response: We thank the reviewer for their comments and have now shared all scripts via a permanent link: https://doi.org/10.5281/zenodo.19422005

Reviewer #2 (Remarks to the Author):

The authors have clearly invested a great deal of effort in revising the manuscript, and it is considerably strengthened. In particular, expanding beyond proteomics to include additional phenotypes (NMR metabolomics and multiple clinical biomarkers, including glycemic traits and lipid-related measures) substantially improved the characterization of the clusters. It was also appreciated that the authors added to the discussion a comparison of the present study to prior work investigating different obesity profiles in relation to T2D risk.

While the manuscript involves well-performed analyses and methodological rigor, it would be ideal for the following two goals to be accomplished 1) mechanistic/molecular interpretability of the clusters (i.e. what pathways(s) are being captured?) and 2) clinical/pathophysiological interpretability (i.e. are these distinct clinical subtypes?). Both are of these are addressed to some extent, and the new analyses are very helpful toward these goals, but I still came away from reading the manuscript unsure as to what the clusters truly represented mechanistically and clinically. For example, how is Cluster 1 meaningfully different from Cluster 2?

Response: We thank the reviewer for this thoughtful comment and fully agree that mechanistic and clinical interpretability are key goals but also challenging in this context. Importantly, we would like to emphasize that we do not expect to identify fully distinct or discrete biological pathways underlying each cluster. The genetic variants included in our analyses represent a spectrum of effects, ranging from harmful to protective, and act through a wide range of biological mechanisms. Consequently, the clustering should not be interpreted as defining four homogeneous and clearly separable groups of variants, but rather to capture dominant patterns within a highly complex and continuous landscape. For this reason, we do not expect complete separation between clusters. For example, the difference between Cluster 1 and Cluster 2

should be interpreted more as a gradient than as a strict qualitative distinction. The purpose of the clustering approach is therefore to reduce the complexity arising from hundreds of independent genetic effects, each potentially acting through different genes and pathways, some overlapping and some distinct. We have now further clarified this point in the Discussion.

While previous studies have often emphasized clustering (typically of patients rather than genetic variants) to define clinically actionable subtypes, our results instead highlight the underlying biological complexity. Individuals are unlikely to fall into clearly distinct categories, and there is likely a continuum of risk profiles rather than discrete subgroups. Nevertheless, even within this continuum, grouping individuals based on shared genetic patterns may still be informative at the population level, where average differences between groups could have implications for risk stratification, prevention, or treatment strategies.

With this said, we do not want to overstate the interpretability of the identified clusters. However, in agreement with the reviewer's suggestion, we have aimed to better summarize the mechanistic and clinical relevance of the clusters in the Discussion and have also added additional analyses as outlined below.

It is possible that consideration of other key physiological or anatomical dimensions of obesity heterogeneity (e.g., body fat distribution and ectopic fat measures, imaging-derived traits, or more direct indices of insulin resistance and beta-cell function) could help. A more systematic phenome-wide characterization might help clarify whether the identified clusters correspond to distinct clinical patterns and ideally might help further differentiate Cluster 1 and 2.

Response: While the manuscript is already comprehensive, we have included a minimal set of additional analyses incorporating imaging-derived traits, including measures of body fat distribution, ectopic fat, and organ volumes. However, these analyses did not further differentiate Cluster 1 and Cluster 2 (Supplementary Figure S6).

Regarding longitudinal data, the authors' point that longitudinal molecular datasets with sufficient sample size are currently limited is well-taken. However, it may be possible to look at longitudinal clinical follow-up (even without repeated molecular profiling), for example how cluster-specific PRS are associated with trajectories of glycemic measures, weight, or incident cardiometabolic outcomes. Such analyses might further strengthen the translational relevance and help support the broader claims about divergent pathways from obesity to T2D. These analyses are not critical, but are just suggestions if they might help paint a clearer picture as to what these clusters represent clinically.

Response: We agree with the reviewer that other clinical outcomes are relevant, and we apologize if we misunderstood this request in the previous revision. In addition to adding imaging-derived traits to the cluster-specific MR

analyses (as described above), we have now also investigated the effect of cluster-specific PRSs in relation to incident cardiometabolic outcomes, including chronic kidney disease, stroke, and coronary artery disease. The results are shown together with the results for type 2 diabetes in Figure 3a.

Overall, the revision substantially strengthens the work and resolves several of the concerns raised previously. The remaining (unfortunately large) limitation is that the clusters are still challenging to fully interpret in terms of both the pathways/disease mechanisms they capture and any clinical application/relevance (particularly with regard to understanding how Cluster 1 differs from Cluster 2).

Response: We thank the reviewer for acknowledging the improvements made in the revision. We fully agree that mechanistic and clinical interpretation of the clusters remains challenging, particularly in distinguishing Cluster 1 from Cluster 2. As noted above, the genetic variants contributing to the clusters represent a spectrum of effects, and the clustering captures dominant patterns within a complex and continuous landscape rather than fully discrete entities. Consequently, the difference between Cluster 1 and Cluster 2 should be interpreted more as a gradient than as a strict qualitative distinction. We have further clarified this in the Discussion and included additional analyses, as suggested by the reviewer, but also addressed the limitation in the Discussion of the revised manuscript.
